# Single-cell RNA sequencing reveals the impact of chromosomal instability on glioblastoma cancer stem cells

**DOI:** 10.1186/s12920-019-0532-5

**Published:** 2019-05-31

**Authors:** Yanding Zhao, Robert Carter, Sivaraman Natarajan, Frederick S. Varn, Duane A. Compton, Charles Gawad, Chao Cheng, Kristina M. Godek

**Affiliations:** 1 Department of Molecular and Systems Biology, Geisel School of Medicine at Dartmouth, Hanover, NH USA; 2 Departments of Oncology and Computational Biology, St. Jude Children’s Research Hospital, Memphis, TN USA; 3 Department of Biochemistry and Cell Biology, HB7200, Geisel School of Medicine at Dartmouth, Hanover, NH 03755 USA; 4 Norris Cotton Cancer Center, Geisel School of Medicine at Dartmouth, Lebanon, NH USA; 5 Department of Biomedical Data Science, Geisel School of Medicine at Dartmouth, Lebanon, NH 03756 USA; 6 Present Address: Jackson Laboratory for Genomic Medicine, Farmington, CT USA; 7 Present Address: Baylor College of Medicine, Houston, TX USA

**Keywords:** Glioblastoma, Cancer stem cells, CSCs, Chromosomal instability, CIN, Aneuploidy, Copy number variation, CNV, Heterogeneity

## Abstract

**Background:**

Intra-tumor heterogeneity stems from genetic, epigenetic, functional, and environmental differences among tumor cells. A major source of genetic heterogeneity comes from DNA sequence differences and/or whole chromosome and focal copy number variations (CNVs). Whole chromosome CNVs are caused by chromosomal instability (CIN) that is defined by a persistently high rate of chromosome mis-segregation. Accordingly, CIN causes constantly changing karyotypes that result in extensive cell-to-cell genetic heterogeneity. How the genetic heterogeneity caused by CIN influences gene expression in individual cells remains unknown.

**Methods:**

We performed single-cell RNA sequencing on a chromosomally unstable glioblastoma cancer stem cell (CSC) line and a control normal, diploid neural stem cell (NSC) line to investigate the impact of CNV due to CIN on gene expression. From the gene expression data, we computationally inferred large-scale CNVs in single cells. Also, we performed copy number adjusted differential gene expression analysis between NSCs and glioblastoma CSCs to identify copy number dependent and independent differentially expressed genes.

**Results:**

Here, we demonstrate that gene expression across large genomic regions scales proportionally to whole chromosome copy number in chromosomally unstable CSCs. Also, we show that the differential expression of most genes between normal NSCs and glioblastoma CSCs is largely accounted for by copy number alterations. However, we identify 269 genes whose differential expression in glioblastoma CSCs relative to normal NSCs is independent of copy number. Moreover, a gene signature derived from the subset of genes that are differential expressed independent of copy number in glioblastoma CSCs correlates with tumor grade and is prognostic for patient survival.

**Conclusions:**

These results demonstrate that CIN is directly responsible for gene expression changes and contributes to both genetic and transcriptional heterogeneity among glioblastoma CSCs. These results also demonstrate that the expression of some genes is buffered against changes in copy number, thus preserving some consistency in gene expression levels from cell-to-cell despite the continuous change in karyotype driven by CIN. Importantly, a gene signature derived from the subset of genes whose expression is buffered against copy number alterations correlates with tumor grade and is prognostic for patient survival that could facilitate patient diagnosis and treatment.

**Electronic supplementary material:**

The online version of this article (10.1186/s12920-019-0532-5) contains supplementary material, which is available to authorized users.

## Background

Intra-tumor heterogeneity contributes to both therapeutic resistance and relapse and poses a major challenge to overcome in the successful treatment of cancers. Intra-tumor heterogeneity stems from diverse populations of cells co-existing within the same tumor that have genetic, epigenetic, functional, and environmental differences [1–3]. Cancer stem cells (CSCs) (also referred to as tumor-initiating cells) are a source of functional cellular heterogeneity in tumors. According to the CSC model, CSCs are at the apex of a functional cellular hierarchy and are the sub-population of cells responsible for tumor initiation and for sustaining tumorigenesis while the population of non-CSCs are non-tumorigenic [3, 4]. Importantly, CSCs contribute to therapeutic resistance and tumor relapse [5, 6].

An additional source of intra-tumor heterogeneity is genetic heterogeneity resulting from DNA sequence variation and/or whole chromosome and focal copy number variations (CNVs). Whole chromosome CNVs are generated by aneuploid or chromosomally unstable populations of tumor cells that have abnormal numbers of chromosomes [7]. Aneuploidy is a stable state with aneuploid cells in a tumor having the same abnormal karyotype, and it is prevalent in cancers with over 90% of solid tumors reported to be aneuploid [8]. In addition, many aneuploid tumor cells also exhibit chromosomal instability (CIN). CIN is a persistent and a high rate of chromosome mis-segregation that causes random chromosome losses and/or gains [7]. Importantly, both CSCs and non-CSCs display CIN [9]. Indeed, we previously demonstrated that for some glioblastoma CSCs with a CIN phenotype each cell in the population had a different karyotype [9]. Thus, despite CSC functional similarity in driving tumorigenesis, CSCs are genetically heterogeneous with diverse karyotypes [9]. Overall, aneuploidy and CIN generate genetic diversity among tumor cells that contributes to therapeutic resistance and is correlated with poor patient prognosis [10–12].

Whole chromosome CNVs, due to aneuploidy, and focal CNVs are also thought to cause alterations in gene transcription [13–18]. Previous studies in aneuploid yeast and mammalian cells demonstrated that the expression level of most genes scaled with chromosome copy number [14–17]. Thus, a change in chromosome copy number due to aneuploidy causes a corresponding change in the transcription levels of most genes on that chromosome. However, these prior studies were performed on stable aneuploid (i.e. all cells in the population had the same abnormal karyotype) and/or genetically selected cells with specific chromosome gains. Furthermore, gene expression measurements were performed on bulk populations, which both homogenizes single cell variation and averages gene expression levels in a population. Accordingly, the relationship between gene expression and chromosome copy number in cells that exhibit CIN, with chromosome complements continuously fluctuating from individual cell to individual cell, remains unexplored.

Here we investigate the impact of CNV due to CIN on gene expression by analyzing the transcriptomes of a glioblastoma cancer stem cell (CSC) line, GliNS2 CSCs, that is chromosomally unstable and a control normal, diploid neural stem cell (NSC) line, CB660 NSCs [9, 19, 20]. We chose to compare NSCs and glioblastoma CSCs because glioblastoma is one of the most lethal cancers [21], and experimental evidence shows that glioblastoma CSC populations are both responsible for tumor development and are resistant to current treatments [5, 22–25]. Thus, there is a critical need to develop new therapeutic strategies that selectively eradicate glioblastoma CSCs but spare normal neural cells. Previous gene expression analysis of CB660 and GliNS2 cells was performed on bulk populations of cells; however, these approaches homogenize the contribution of single cell CNV to gene expression levels and to differentially expressed genes between NSCs and glioblastoma CSCs [20, 26]. Only single-cell methods are suitable to determine the impact of CNV due to CIN on gene expression. Accordingly, we performed single-cell RNA sequencing of individual CB660 NSCs and GliNS2 CSCs to investigate the influence of CNV on gene expression levels in chromosomally unstable cells and to investigate the contribution of CNV to gene expression differences between NSCs and glioblastoma CSCs.

## Results

### Neural stem cells and glioblastoma cancer stem cells have distinct transcriptomes

**Fig. 1:**
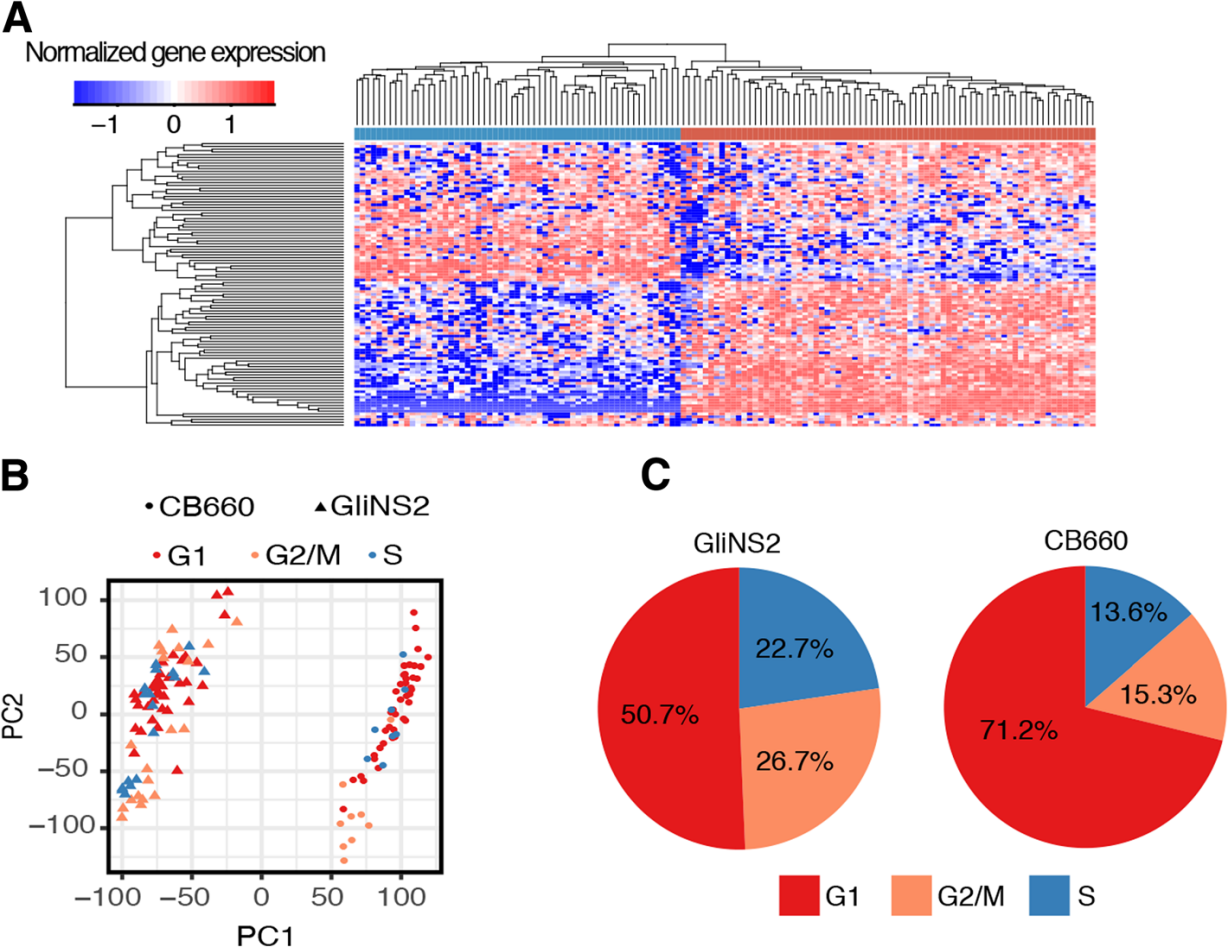
CB660 NSCs and GliNS2 CSCs have distinct gene expression profiles. **a**, Heatmap showing the normalized gene expression of the most variably expressed genes used for hierarchical clustering analysis. The top dendrogram shows that CB660 NSCs and GliNS2 CSCs cluster into two distinct populations. In the row above the heatmap, blue indicates CB660 NSCs and red indicates GliNS2 CSCs. **b**, Principal component analysis (PCA) and cell cycle phase analysis of CB660 NSCs and GliNS2 CSCs. The graph shows the separation of CB660 NSCs (circles) and GliNS2 CSCs (triangles) into distinct groups. The color of the circles or triangles corresponds to the predicted cell cycle phase of each cell. **c**, Pie plots showing the fraction of CB660 NSCs and GliNS2 CSCs in each phase of the cell cycle. *P* > 0.05; Chi-square test comparing the cell cycle profiles of CB660 NSCs and GliNS2 CSCs

To investigate the relationship between gene expression levels and CNV in chromosomally unstable cells, we performed single-cell RNA sequencing of chromosomally unstable GliNS2 glioblastoma CSCs and control normal, diploid CB660 NSCs that were grown in identical serum-free culture conditions [9, 19, 20]. After performing data normalization and filtering steps, we obtained high quality data for 59 CB660 NSCs and 75 GliNS2 CSCs (Additional file 1a-e). As an initial comparison of CB660 NSCs and GliNS2 CSCs, we performed unsupervised hierarchical clustering using the most variably expressed genes. Hierarchical clustering showed that CB660 NSCs and GliNS2 CSCs cluster into two distinct groups as expected (Fig. 1a). As further validation, we performed principal component analysis (PCA), and similar to the unsupervised hierarchical clustering, we found that CB660 NSCs and GliNS2 CSCs separated into two distinct groups (Fig. 1b). In addition, we used gene expression profiles to computationally infer the cell cycle phase of each cell. Specifically, we utilized previously published methods that combine a set of cell cycle annotated genes with a pair-based classifier to assign cell cycle phases [27, 28]. CB660 NSCs and GliNS2 CSCs had different percentages of cells in each phase of the cell cycle, but overall these differences were not statistically significant (Fig. 1b-c).

### Chromosomal instability affects gene expression levels

Since the technology to perform simultaneous single-cell DNA sequencing to obtain the copy number profile and single-cell RNA sequencing to obtain the gene expression profile of the same cell is not readily feasible, we used gene expression levels to computationally infer large-scale CNV in chromosomally unstable GliNS2 CSCs [29]. The approach is based upon a previously published method that averages gene expression over large genomic regions to infer whole chromosome copy number alterations [29]. Importantly, the method requires gene expression measurements from control diploid cells to establish reference gene expression levels [29]. More specifically, we calculated GliNS2 CSC relative gene expression levels against reference normal, diploid CB660 NSC gene expression and then determined the copy number of a gene as the average relative expression of its neighboring 50 downstream genes and 50 upstream genes. We repeated this iteratively to predict the copy number of large genomic regions. Initially, we validated this method using matched copy number and gene expression data from glioblastoma multiforme (GBM) and breast invasive carcinoma (BRCA) samples in The Cancer Genome Atlas (TCGA). As expected, there was significantly more inferred CNV in both GBM and BRCA tumor samples compared to normal samples with a median Spearman correlation = 0.43 or = 0.46 between the estimated CNV and known CNV across all GBM and BRCA tumor samples, respectively (Additional file 2 a-d and Additional file 3 a-d). As further validation, we calculated a chromosomal instability index to quantify CNV by determining the average absolute estimated copy number (ECN) of each tumor sample, and as expected, both GBM and BRCA tumor samples had a significant increase in the chromosomal instability index compared to normal samples (Additional file 2b and Additional file 3b). Also, the BRCA tumor samples had a significantly higher median chromosomal instability index compared to the GBM tumor samples (BRCA median = 0.069, GBM median = 0.056, *p* < 0.05) in agreement with BRCA tumors having a higher aneuploidy score than GBM tumors [30].

**Fig. 2:**
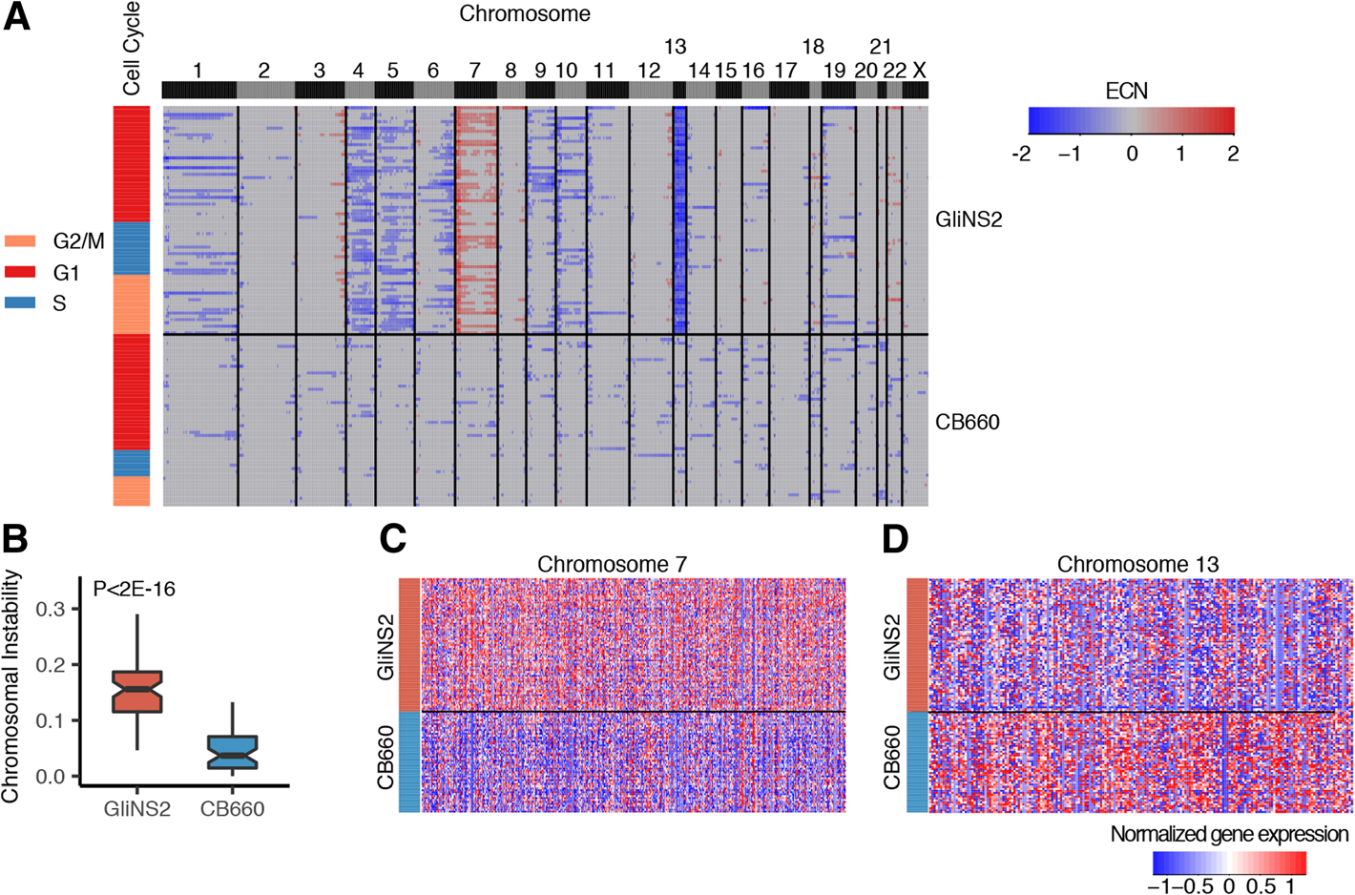
Gene expression scales with chromosome copy number. **a**, Heatmap of the estimated copy number (ECN) of all chromosomes (columns) in single CB660 NSCs and GliNS2 CSCs (rows). On the scale, ECN = 0 indicates diploid gene expression levels. The column adjacent to the heatmap shows the cell cycle phase of each cell as determined in Fig. 1b with the color of the bar corresponding to the predicted cell cycle phase. **b**, Quantification of chromosomal instability in CB660 NSCs and GliNS2 CSCs. Bar, median; box 25th to 75th percentile; whiskers, minimum and maximum. *P* < 2E-16; Mann-Whitney U test comparing CB660 NSCs and GliNS2 CSCs. **c** and **d,** Heatmaps showing normalized gene expression in CB660 NSCs and GliNS2 CSCs for chromosome 7 (C) and chromosome 13 (D)

Next, we applied this method to our single-cell RNA sequencing data. Based upon consideration for the number of genes located on each chromosome and filtering out genes with low expression, we used 6350 genes that represent the top 30th percentile of genes across all samples (Additional file 4a). Also, we applied an additional filtering step for cells with low expression of the 6350 genes using a cutoff of low normalized library size < 26,000 counts or a high fraction of zero counts (< 25%) (Additional file 4a). After applying the additional filtering step, 52 CB660 NSCs and 69 GliNS2 CSCs remained for analysis. Previously, we showed that CB660 NSCs are predominantly diploid [9], so we averaged the expression of each of the 6350 genes across CB660 NSCs to establish reference normal, diploid gene expression levels for comparison to the gene expression profiles of single cells. The ECN profiles for single cells showed more copy number alterations in GliNS2 CSCs compared to CB660 NSCs (Fig. 2a). Accordingly, GliNS2 CSCs had a significantly higher chromosomal instability index compared to CB660 NSCs (Fig. 2b). Furthermore, there was no correlation between the inferred CNV and cell cycle phase for either CB660 NSCs or GliNS2 CSCs (Fig. 2a). Overall, these results demonstrate that, as expected of chromosomally unstable cells, GliNS2 CSCs have significantly more CNV compared to normal, diploid CB660 NSCs.

To validate this approach, we shuffled neighboring genes across the genome so that the gene expression for each gene was not associated with its position on a chromosome and repeated the copy number and chromosomal instability estimation analyses. After shuffling neighboring genes, no CNV pattern was observed for the GliNS2 CSCs, and moreover, there was no significant difference in the chromosomal instability index of CB660 NSCs and GliNS2 CSCs (Additional file 4b-c). Also, we tested the requirement for a normal, diploid reference to accurately determine CNV by performing our ECN analysis using the average expression of GliNS2 CSCs as the reference (Additional file 4d). This approach did not detect common chromosome copy number alterations such as the gains in chromosome 7 or the loss of chromosome 13 (Fig. 2a) because there is minimal fold change between the average reference gene expression levels and the gene expression levels in single GliNS2 CSCs.

In addition to the increased CNV in GliNS2 CSCs compared to CB660 NSCs, the ECN analysis also revealed karyotype heterogeneity among single GliNS2 CSCs as shown by the cell-to-cell variation in chromosome losses and gains (Fig. 2a) and the range of total CNV among GliNS2 CSCs as measured by the chromosomal instability index (Fig. 2b). For example, chromosome 9 and 10 loss are commonly reported in GBM [29, 31], and in agreement, our ECN analysis identified chromosome 10 loss in the majority of TCGA GBM tumor samples sequenced using bulk methods (Additional file 2a); however, single GliNS2 CSCs showed variation with respect to chromosome 9 or 10 copy numbers. Indeed, only 30.4% or 27.5% of GliNS2 CSCs had an ECN loss for chromosome 9 or 10, respectively (Fig. 2a). In support, previous cytogenomic profiles of GliNS2 CSCs also showed heterogeneity among single cells with both gains and losses in chromosome 9 and 10 reported [9, 32]. Overall, our estimated copy number analysis demonstrates that GliNS2 CSCs have extensive genetic heterogeneity with respect to chromosome copy numbers in agreement with our previous karyotyping data showing that no two GliNS2 CSCs have the same karyotype [9]. Also, these results demonstrate the utility of single-cell vs. bulk population approaches when analyzing chromosomally unstable cells.

Although GliNS2 CSCs had extensive karyotype heterogeneity, there were examples of chromosome copy number alterations that occurred in the majority of GliNS2 CSCs. We utilized these common alterations, to determine the impact of whole chromosome CNV due to CIN on gene expression. Specifically, we compared the ECNs for chromosomes 7 and 13 to previous karyotyping data [9]. because the majority of GliNS2 CSCs were predicted to have gained or lost copies of chromosome 7 and 13, respectively. The estimated copy number analysis predicted 4 or more copies for most of chromosome 7 in the majority of GliNS2 CSCs (Fig. 2a). This estimated copy number is in agreement with previously published spectral karyotyping data that demonstrated 76% of GliNS2 CSCs had 4 or more copies of chromosome 7 with a chromosome mode of 6 copies [9]. In addition, a comparison of gene expression profiles for chromosome 7 showed that 83.9% of genes were up-regulated in GliNS2 CSCs compared to CB660 NSCs (Fig. 2c). Further, the estimated copy number analysis predicted a loss of chromosome 13 in GliNS2 CSCs (Fig. 2a). In agreement, previous spectral karyotyping data showed that 36% of GliNS2 CSCs had 1 or 0 copies of chromosome 13 [9]. Also, a comparison of gene expression profiles for chromosome 13 showed that in GliNS2 CSCs 79.6% of genes were down-regulated compared to CB660 NSCs (Fig. 2d). Overall, these results demonstrate that gene expression scales with chromosome copy number for the majority of genes on a given chromosome in chromosomally unstable cells.

Although for chromosomes 7 and 13 the estimated copy numbers from the gene expression data were in agreement with previous karyotyping data, not all predicted CNV in GliNS2 CSCs was detected. For example, previous spectral karyotyping demonstrated that 84% of GliNS2 CSCs had 3 or more copies of chromosome 12 with a chromosome mode of 3 copies [9], but the analysis did not identify any GliNS2 CSCs with 3 or more copies of chromosome 12 (Fig. 2a). One explanation is that the entire population of GliNS2 CSCs analyzed had 2 copies of chromosome 12 since cells were sequenced at random. Alternatively, there may be compensatory mechanisms that buffer gene expression levels on chromosome 12 against chromosome CNV. Although a previous study reported gene-dosage compensation in aneuploid yeast cells, only 10–30% of genes on a given chromosome were buffered against copy number changes [33]. Lastly, there may be limitations on the sensitivity of computationally inferring copy number from single-cell gene expression data. In comparison, the gain in chromosome 7 copies is at a minimum a 2-fold increase in expression levels from genes on that chromosome while an increase from two to three copies of chromosome 12 is only a 1.5-fold change.

### CNV dependent and independent mechanisms contribute to differential gene expression

**Fig. 3:**
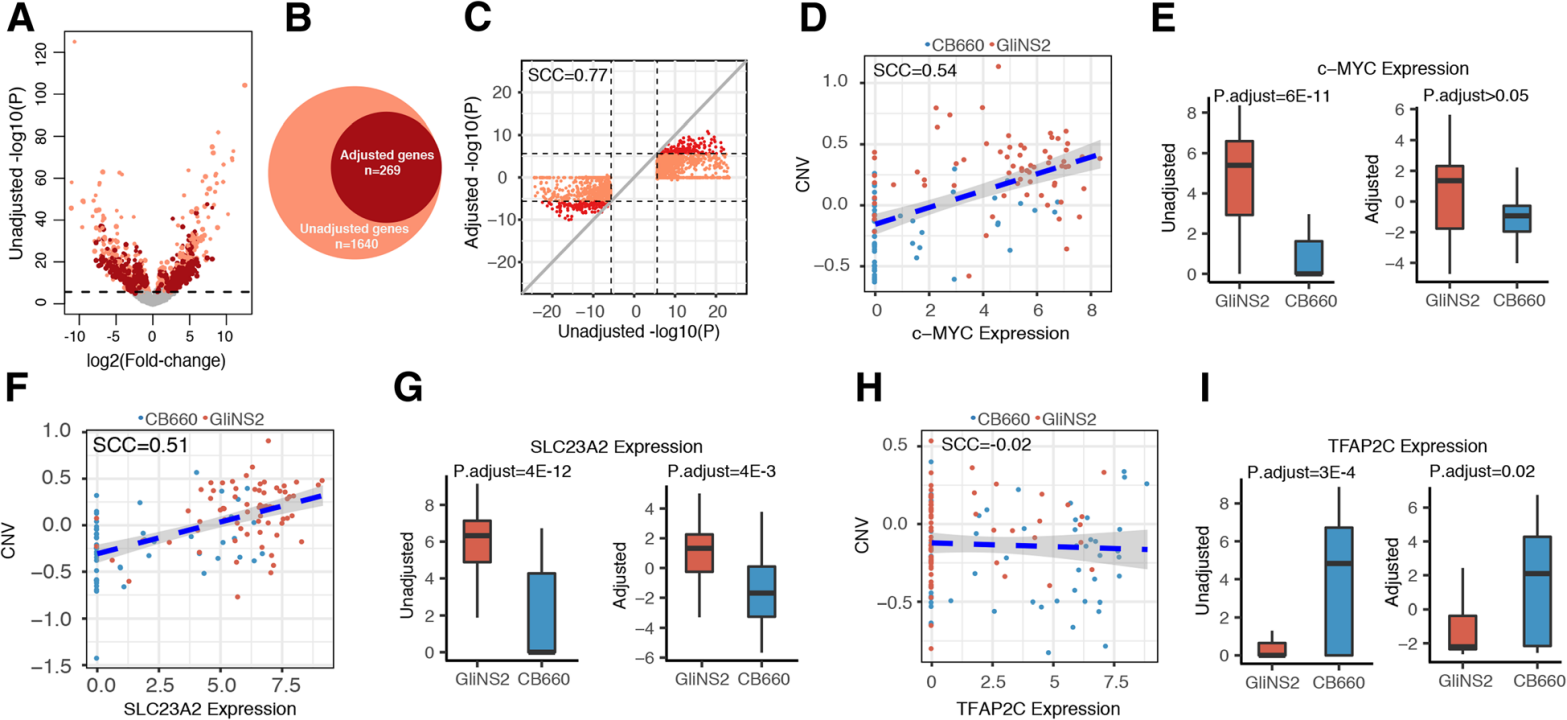
Identification of copy number dependent and independent differentially expressed genes between CB660 NSCs and GliNS2 CSCs. **a**, Volcano plot showing both unadjusted and copy number adjusted differentially expressed genes between CB660 NSCs and GliNS2 CSCs**.** The dashed line shows the statistical significance cut-off (P.adjust< 0.05, Mann-Whitney U test and Bonferroni adjustment) used for differential gene expression analysis. Dark red points indicate genes that remain significantly differentially expressed after copy number adjustment while light red points indicate genes that are not significantly differentially expressed after copy number adjustment. **b**, Venn diagram showing the number of overlapping differentially expressed unadjusted and copy number adjusted genes. **c**, Scatter plot showing the correlation between the negative log10 transformed *p*-value of unadjusted and copy number adjusted differential gene expression analysis. Spearman correlation coefficient = 0.77. **d**, Scatter plot showing the correlation between c-MYC expression and c-MYC copy number in CB660 NSCs and GliNS2 CSCs. Spearman correlation coefficient = 0.54. **e**, Bar graphs quantifying c-MYC expression in CB660 NSCs and GliNS2 CSCs before (left graph) and after copy number adjustment (right graph). Bar, median; box 25th to 75th percentile; whiskers, minimum and maximum. P.adjust> 0.05 after copy number adjustment, Mann-Whitney U test and Bonferroni adjustment. **f**, Scatter plot showing the correlation between SLC23A2 expression and SLC23A2 copy number in CB660 NSCs and GliNS2 CSCs. Spearman correlation coefficient = 0.51. **g**, Bar graphs quantifying SLC23A2 expression in CB660 NSCs and GliNS2 CSCs before (left graph) and after copy number adjustment (right graph). Bar, median; box 25th to 75th percentile; whiskers, minimum and maximum. P.adjust = 4E-3 after copy number adjustment, Mann-Whitney U test and Bonferroni adjustment. **h**, Scatter plot showing the correlation between TFAP2C expression and TFAP2C copy number in CB660 NSCs and GliNS2 CSCs. Spearman correlation coefficient = − 0.02. **i**, Bar graphs quantifying TFAP2C expression in CB660 NSCs and GliNS2 CSCs before (left graph) and after copy number adjustment (right graph). Bar, median; box 25th to 75th percentile; whiskers, minimum and maximum. P.adjust = 0.02 after copy number adjustment, Mann-Whitney U test and Bonferroni adjustment

In addition to investigating the impact of whole chromosome CNV due to CIN on gene expression, we also performed differential gene expression analysis between CB660 NSCs and GliNS2 CSCs to discover new insights into CSCs biology. In total the expression of 1640 genes was significantly increased or decreased in GliNS2 CSCs compared to CB660 NSCs (Fig. 3a) (Additional file 8: Table S1). Multiple biological mechanisms may account for gene expression differences between CB660 NSCs and GliNS2 CSCs including CNV due to either whole chromosome and/or focal copy number alterations. After performing differential gene expression analysis between CB660 NSCs and GliNS2 CSCs, we then determined the contribution of CNV to the expression levels of these differentially expressed genes. To do this, we estimated the copy number of each differentially expressed gene and then adjusted individual gene expression levels taking into account the inferred copy number. Initially, we validated our approach by comparing gene expression levels adjusted for copy number using either SNP-array measured CNV or RNA-seq inferred CNV in TCGA GBM tumor samples. There was good agreement between the two approaches with a median correlation = 0.92 (Additional file 5a). Next, we predicted the copy number of individual differentially expressed genes that were included in our initial analysis of 6350 genes by determining the copy number of a gene as the average relative expression of its neighboring 50 downstream genes and 50 upstream genes as described above. For the remaining differentially expressed genes, we predicted their copy number based upon their nearest neighbor in the group of 6350 genes. Notably, this approach will include both CNV arising from whole chromosome or focal alterations.

On a global scale, the mean log2 fold change in expression for the majority of differentially expressed genes shifted after copy number adjustment (Additional file 5b). Importantly, after adjusting individual gene expression levels for copy number, the differential expression of 1360 genes was no longer statistically significant (Fig. 3a-c) (Additional file 8: Table S2). The statistical significance of each gene’s contribution to the difference between GliNS2 CSCs and CB660 NSCs decreased after their copy number adjustment, indicating that CNV accounts for the majority of the transcription level difference at the single cell level (Additional file 8: Table S2). Moreover, though the negative log10 transformed *p* values before and after adjustment were highly correlated, the inconsistency between them indicated that some genes are more affected by CNV than other genes (Fig. 3c). For example, the transcription factor c-MYC is an individual gene whose expression illustrated copy number dependent differential gene expression. C-MYC expression positively correlated (SCC = 0.54) with inferred copy numbers in CB660 NSCs and GliNS2 CSCs (Fig. 3d). Expression analysis unadjusted for copy number demonstrated that c-MYC expression was significantly increased in GliNS2 CSCs compared to CB660 NSCs; however, after adjusting for copy number the estimated expression of c-MYC was no longer significantly different between GliNS2 CSCs and CB660 NSCs (Fig. 3e). In addition, there were examples of genes that had significantly increased expression levels in CB660 NSCs compared to GliNS2 CSCs when unadjusted for copy number; however, after copy number adjustment, the gene expression levels were no longer significantly different (Additional file 8: Table S2). Thus, these results demonstrate that the gene expression differences between CB660 NSCs and GliNS2 CSCs for most genes can be accounted for through CNV due to either whole chromosome or focal alterations.

On the other hand, after adjusting for copy number, the differential expression of 269 genes remained significantly different between CB660 NSCs and GliNS2 CSCs (Fig. 3b-c) (Additional file 8: Table S3). Within the 269 copy number independent differentially expressed gene set there were genes whose expression was influenced by copy number yet their expression remained significantly different between CB660 NSCs and GliNS2 CSCs even after adjusting gene expression levels for copy number. An example is SLC23A2 whose gene expression level positively correlated (SCC = 0.51) with inferred copy numbers in CB660 NSCs and GliNS2 CSCs (Fig. 3f). Yet, after adjusting for copy number, the expression of SLC23A2 remained significantly increased in GliNS2 CSCs compared to CB660 NSCs (Fig. 3g). In total, only 25% (Spearman correlation ≥0.3) of the copy number independent genes had expression levels that were positively influenced by estimated copy number but still remained significantly differentially expressed after adjusting gene expression levels for copy number. These results demonstrate that for some of the copy number independent differentially expressed genes, multiple mechanisms regulate gene expression levels, including copy number, to contribute to the total level of differential expression between CB660 NSCs and GliNS2 CSCs.

Yet for the majority of genes in the copy number independent differentially expressed gene set there was virtually no change in differential gene expression levels after adjusting for copy number. An example of this is the transcription factor TFAP2C whose gene expression level did not correlate (SCC = -0.02) with inferred copy numbers in CB660 NSCs and GliNS2 CSCs (Fig. 3h), and after adjusting for copy number the estimated expression of TFAP2C remained significantly decreased in GliNS2 CSCs (Fig. 3i). In addition, there were examples of genes whose expression remained significantly increased in GliNS2 CSCs even after adjusting gene expression levels for copy number (Additional file 8: Table S3). These results demonstrate that for most copy number independent differentially expressed genes transcriptional and/or post-transcriptional mechanisms predominantly regulate their gene expression with no or minimal contribution of copy number to the total level of differential expression between CB660 NSCs and GliNS2 CSCs.

### A CNV independent gene signature correlates with tumor grade and is prognostic for patient survival

**Fig. 4:**
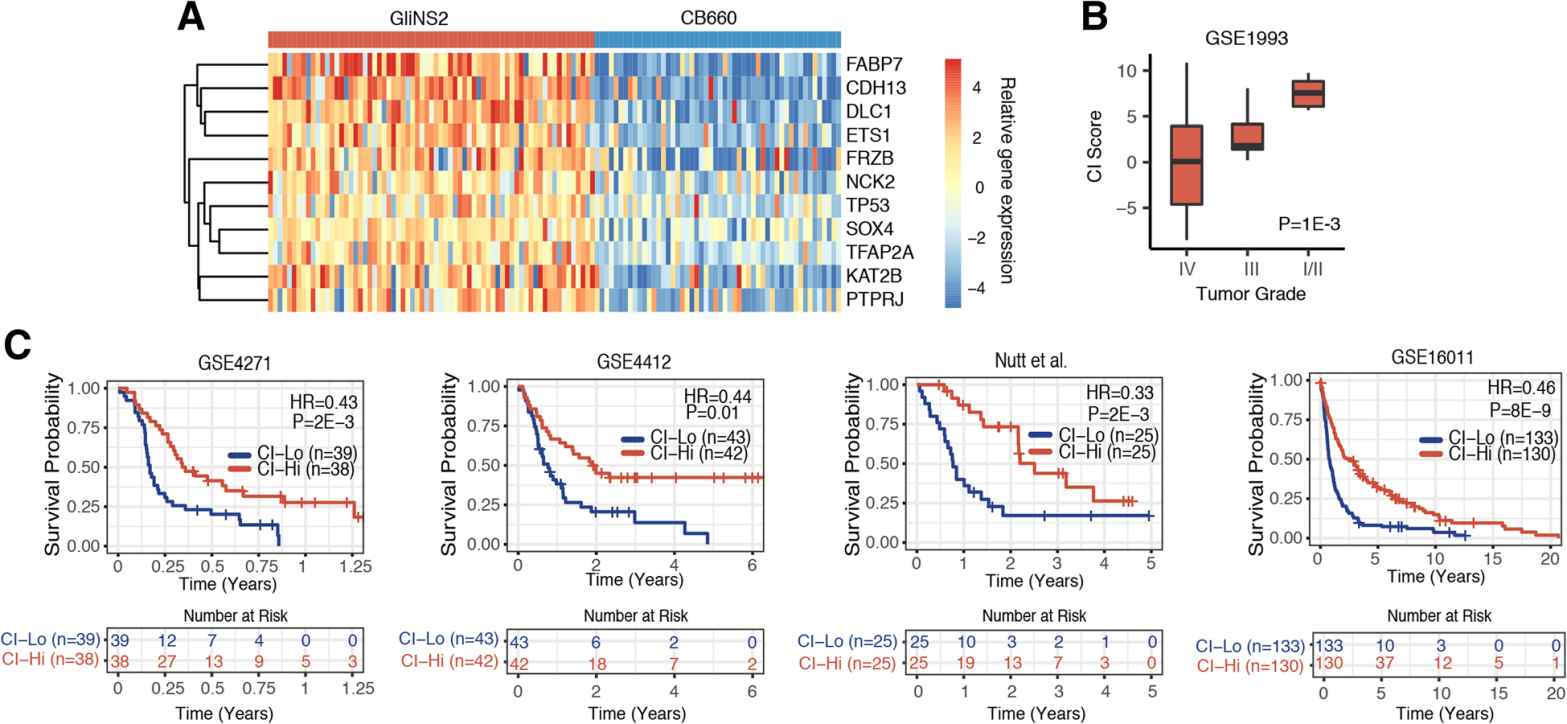
A copy number independent gene signature is prognostic for tumor grade and patient survival. **a**, Heatmap showing the relative gene expression of the copy number independent differentially expressed genes that are involved in pathways that negatively regulate cell proliferation in GliNS2 CSCs compared to CB660 NSCs. **b**, Graph showing the copy number independent (CI) gene signature score for glioma samples in data set GSE1993 stratified by histological grade. Grade I/II = pilocytic astrocytoma (*n* = 2) and diffuse astrocytoma (*n* = 5), Grade III = anaplastic astrocytoma (*n* = 19), and Grade IV = glioblastoma (*n* = 39). Bar, median; box 25th to 75th percentile; whiskers, minimum and maximum. *P* = 1E-3, ANOVA analysis. **c**, Kaplan-Meier plots showing that the CI gene signature score is prognostic for patient survival in four independent data sets. The median CI score was used as the cutoff to dichotomize patients into CI-Hi and CI-Lo groups with the number of patients in each group indicated in parentheses. Hazard ratios were calculated using a Cox regression model, and *p*-values were calculated by using log-rank tests to determine statistical differences between survival curves. Below each Kaplan-Meier plot is a table showing the number of patients at risk over time

We focused our further analysis efforts on the 269 copy number independent differentially expressed genes for the following reason: these genes are differentially expressed between normal stem cells and CSCs, but importantly, this gene set may also offer insights into mechanisms that allow CSCs to maintain functional similarity despite a CIN phenotype with continual fluctuations in chromosome copy numbers. First, we performed Gene Ontology (GO) enrichment analysis on both the copy number independent significantly up- and down-regulated differentially expressed genes (Fig. 4a, Additional file 8: Table S3, Additional file 6a-b, and Additional file 8: Table S4). For the genes with increased expression levels independent of copy number in GliNS2 CSCs relative to CB660 NSCs, the most significantly enriched pathway involved the negative regulation of cell proliferation (Fig. 4a and Additional file 6a). In this category were genes that regulate several major signaling pathways that control proliferation including the secreted frizzled related protein, FRZB (also known as sFRP3), that is an antagonist of the Wnt pathway [34], the protein tyrosine phosphatase, PTPRJ, that negatively regulates tyrosine kinase signaling [35], and the GTPase activating protein, DLC-1, that inhibits Rho signaling [36]. (Fig. 4a and Additional file 8: Table S5). Although cancer cells are typically defined by uncontrolled proliferation, previous studies have shown that CSCs are a slow-proliferating/quiescent population of cells in comparison to non-CSCs [22, 37]. In agreement with the slow-proliferating/quiescent phenotype of CSCs [22, 37], GliNS2 CSCs double approximately every 74 h in culture compared to glioma non-CSCs that double between every 21–46 h in culture (Additional file 6c) [20, 38]. Thus, up-regulated expression of these genes may contribute to this phenotype and allow GliNS2 CSCs to maintain a slow-proliferating phenotype despite exhibiting CIN.

Second, from the expression pattern of the copy number independent genes, we established a copy number independent (CI) gene signature. We validated our CI gene signature using publicly available gene expression data to test for association between additional glioma CSCs, glioma non-CSCs, and normal astrocytes with a CI gene signature score [39, 40]. Glioma CSCs had the highest CI gene signature score followed by glioma non-CSCs and then normal astrocytes (Additional file 7a). Thus, the CI gene signature is a gene expression pattern that is enriched in multiple glioma CSCs and is not unique to only GliNS2 CSCs. Also, we tested if the CI gene signature correlated with a previously defined gene signature for glioblastoma proneural, neural, classical, or mesenchymal molecular sub-types [41]. Overall, 34 genes overlapped between the 269-CI gene signature and the 840-gene signature established by Verhaak et al. [41]. Although the proneural molecular sub-type had the highest CI gene signature score (Additional file 7b), a heatmap of the non-overlapping genes showed that the CI gene signature did not clearly distinguish any of the four molecular sub-types (Additional file 7c). Thus, the CI gene signature is a novel set of genes that does not overlap with the previously characterized gene signature for proneural, neural, classical, or mesenchymal glioma molecular sub-types.

To further explore the relevance of the CI genes to the pathogenesis of gliomas, we tested for association between both tumor grade and patient survival time with a score for the CI gene signature. Low grade I/II pilocytic or diffuse astrocytomas tumors scored highest for the CI gene signature followed by grade III anaplastic astrocytomas, and then grade IV glioblastoma tumors (Fig. 4b) [42]. To independently confirm this result, we tested for association of the CI gene signature score with tumor grade using a distinct gene expression data set [43]. This analysis confirmed our finding of a correlation between tumor grade and CI gene signature score (Additional file 6d). These results demonstrate the broad applicability of a CI gene signature score to stratify multiple types of gliomas by tumor grade despite the fact that the GliNS2 CSCs used to derive the CI gene signature were established from a grade IV glioblastoma [20].

Glioma tumor grading is based in part upon histological features assessed by tissue morphology and one criterion is the level of mitotic activity present in specimens with low grade tumors having minimal mitotic activity while high grade tumors have high levels of proliferation [44]. Accordingly, these results show that low grade tumors that proliferate slower had the highest CI gene signature score. One mechanism that may account for this is the increased expression of genes involved in pathways that negatively regulate cell proliferation in the CI gene signature (Fig. 4a).

In addition to tumor grade, we also tested for association between patient survival time with a score for the CI gene signature. We used tumor gene expression data integrated with clinical data, to dichotomize patients into CI-Hi and CI-Lo groups using the median CI score to stratify patients into either group. Patients in the CI-Hi group had a significantly longer survival time than patients in the CI-Lo group (e.g. GSE16011 HR = 0.46, *P* = 8E-9). Overall, the CI gene signature score was prognostic for patient survival (Fig. 4c) in four independent patient data sets [43, 45–48].

Given the correlation between the CI gene signature score and tumor grade (Fig. 4b) and that patients with low grade tumors have significantly increased median survival times compared to patients with high grade gliomas [44], we reasoned that the CI-Lo and CI-Hi groups may also stratify patients by tumor grade. In agreement, there was a significant percent increase in patient samples with grade III tumors in the CI-Hi group compared to the CI-Lo group with only 13.2% of the samples in the CI-Lo group (*n* = 43) having grade III tumors (Additional file 6e) [46]. Taken all together, these results demonstrate that the CI gene signature score defined here is prognostic for glioma tumor grade and patient survival.

## Discussion

### CIN is a source of intra-tumor genetic and transcriptional heterogeneity

Here we show that the genetic diversity generated by CIN also leads to heterogeneity in gene expression levels. In contrast to a stable aneuploid state, CIN is a persistent source of change. Indeed, both our previous karyotyping data [9]. and our ECN profiles of single chromosomally unstable CSCs demonstrate the extensive genetic heterogeneity that arises due to CIN. Such heterogeneity necessitates the use of single-cell methods to investigate the impact of genetic diversity on tumor cell functions; however, single-cell multiomics approaches to simultaneously determine chromosome copy number and additional omics profiles are not currently readily feasible. To overcome this limitation, we used single-cell RNA sequencing combined with bioinformatics analysis to estimate the impact of CNV on gene expression in chromosomally unstable CSCs. Although compared to single-cell DNA-seq or SNP arrays, the resolution of this method is more limited to detect CNV, this approach importantly does not require performing simultaneous copy number and gene expression sequencing on the same single cell.

Moreover, we show that in chromosomally unstable CSCs whole chromosome CNV due to CIN generates a corresponding change in transcript levels for the majority of genes on a given chromosome. Further, our data demonstrate that both chromosome loss or chromosome gains cause a corresponding decrease or increase, respectively, in gene expression levels. To our knowledge, this is the first demonstration of cell-to-cell variation in gene expression caused by changes in chromosome copy number in chromosomally unstable cells. Thus, CIN is not only a source of genetic heterogeneity but also a source of transcriptional heterogeneity because the expression of most genes scales with chromosome copy number in each cell.

Importantly, CIN will cause large-scale transcriptional alterations, as the copy number of numerous genes will change simultaneously following mis-segregation of a whole chromosome. Consequently, the karyotype and transcriptional changes caused by CIN provide one mechanism for studies that infer dosage-sensitive pathways fuel tumor initiation, evolution and adaptability. For example, a previous study proposed that the cumulative gene-dosages of tumor suppressor STOP genes and oncogenic GO genes drive tumorigenesis [49]. Indeed, our data for c-Myc expression levels provides direct evidence that changes in gene expression levels caused by copy number alterations influence the gene dosage of GO genes [49]. Therefore, CIN is one mechanism to alter the gene-dosage of STOP and GO genes giving tumor cells the adaptability needed to survive and propagate under changing selective pressures during tumor growth. Conversely, a direct relationship between chromosome copy number and gene expression levels also provides a mechanism for previous studies proposing to increase the rate of CIN above a tolerable threshold as a therapeutic strategy to cause a loss of tumor cell function and/or viability [9, 50–52]. The opposing effects of CIN may in part depend on the frequency of altering gene expression levels with low rates of change providing adaptability but high rates of change frequently generating transcriptional programs that are incompatible with supporting tumorigenesis or viability [9, 52, 53]. Thus, the genetic and transcriptional diversity caused by CIN can be beneficial or detrimental depending on the rate of mis-segregation.

Previous studies using stable aneuploid yeast and mammalian cells have shown that the levels of most proteins tend to reflect mRNA levels and to scale proportionally with chromosome copy number [16, 17, 54]. Based on those results and our data, we would predict that CIN generates extensive cell-to-cell variation in protein levels in conjunction with karyotype and transcriptional changes arising from the persistent and high rates of chromosome mis-segregation. A direct test of this prediction will require the development of quantitative methodology for the analysis of genome, transcriptome, and proteome levels within single cells.

### Buffering gene expression against CIN

Our data demonstrate that having a CIN phenotype will not only cause continual fluctuations in the karyotypes but also the transcriptomes (and, by extension, the proteomes) of tumor cells. Such continual alterations in chromosome copy numbers and transcriptional programs may influence the functional properties of tumor cells including CSC populations. In support of this, elevated rates of CIN in CSCs drive CSCs to a non-CSC phenotype causing a loss of CSC function and inhibition of tumor initiation [9]. Accordingly, this raises the question of how CSCs maintain functional similarity to drive tumorigenesis despite a CIN phenotype. Our differential gene expression analysis of NSCs and CSCs demonstrates that CNV accounts for most differential gene expression but that the differential expression of a subset of genes is independent of CNV. Consequently, the copy number independent regulation of gene expression buffers transcript levels against continual fluctuations in whole chromosome copy numbers due to CIN and, presumably, against focal copy number alterations as well. Buffering gene expression levels from copy number changes is one mechanism that preserves gene expression patterns and would allow CSCs to maintain functional similarity in the presence of a CIN phenotype. In support, we show that the genes differentially expressed and up-regulated independent of copy number in glioblastoma CSCs compared to normal NSCs are enriched in pathways that negatively regulate cell proliferation. A defining functional characteristic of CSCs is that CSCs are slow-proliferating/quiescent cells [22, 37], and the up-regulated copy number independent expression of these genes may allow glioblastoma CSCs to maintain this phenotype despite continual changes in karyotype and transcriptional programs due to CIN. Importantly, given that CSCs are thought to be resistant to current therapeutics in part because of their slow-proliferating/quiescent phenotype an important implication of this work is that insulating the expression levels of certain genes against CNV may provide a selective advantage to CSCs. Overall, the buffering of gene expression levels against CIN may not be unique to CSCs but rather a general phenomenon that occurs in other chromosomally unstable tumor cells that must cope with continual copy number and transcriptional changes. The experimental interrogation of non-CSCs for genes whose expression levels are regulated independent of copy number will address this possibility.

Multiple cis- or trans-acting effects may buffer gene expression levels against CNVs including DNA sequence mutations and transcriptional or post-transcriptional mechanisms. Transcriptional or post-transcriptional mechanisms include epigenetic mechanisms such as histone modifications or DNA methylation or post-transcriptional mechanisms such as RNA processing that modifies RNA stability. Identification of the specific mechanisms that buffer gene expression levels from CNV opens potential therapeutic opportunities. Given that the regulation of gene expression independent of copy number may allow CSCs to maintain functional similarity, targeting the mechanisms responsible may render their expression copy number-dependent leading to transcriptional programs in CSCs that drive CSCs to a non-CSC phenotype inhibiting tumorigenesis and making these cells more susceptible to treatment.

### Clinical relevance of the CNV independent gene signature

These data not only uncover mechanistic insights into how CSCs maintain functional similarity despite a CIN phenotype, but importantly, the copy number independent differentially expressed gene set also defines a signature that is prognostic for tumor grade and patient survival. Gliomas account for over 70% of malignant brain tumors in adults making gliomas the most common form of primary malignant brain tumors [44, 55]. Gliomas are sub-classified based upon histological and molecular features according to guidelines put forth by The World Health Organization (WHO) [56] with glioblastoma grade IV tumors being the most aggressive and lethal brain tumors. Patients diagnosed with glioblastoma have only a 10% five-year survival rate despite aggressive treatment with radiotherapy and chemotherapy [21]. Extensive experimental evidence shows that glioblastoma CSCs are the population of tumor cells responsible for tumor development and therapy resistance [5, 22–24, 57].

We find that the CI gene signature score derived from differentially expressed genes between normal NSCs and glioblastoma grade IV CSCs stratifies gliomas according to tumor grade with grade I/II tumors scoring the highest followed by grade III tumors, and grade IV glioblastoma tumors scoring the lowest. One criterion for assessing tumor grade is the proportion of proliferating tumor cells found upon histological examination with low grade gliomas being more indolent and less proliferative than high grade gliomas [44, 58]. Our data suggests that the up-regulation of genes that negatively regulate proliferation, including genes that inhibit several major signaling networks controlling cell growth, in the CI gene signature may provide an explanation for the correlation with tumor grade. In addition, patients with low grade gliomas have a significant increase in median survival time compared to patients diagnosed with high grade gliomas that is in part attributed to the levels of mitotic activity present in tumors [44, 58]. Accordingly, we also show that the CI gene signature is prognostic for patient survival with patients in the high CI gene signature score group (CI-Hi) having either grade III or IV tumors and increased survival times compared to patients in the low CI gene signature score group (CI-Lo) having predominantly grade IV tumors and decreased survival times. Overall, the CI gene signature is prognostic for tumor grade and patient survival.

Importantly, the stratification of tumor grade and patient survival by CI gene signature score has clinical implications that could facilitate patient diagnosis and treatment. In clinical practice, tumor grade is assessed morphologically and determined based upon levels of nuclear atypia, mitotic activity, microvascular proliferation, and necrosis; therefore, diagnosis is conditional on observer subjectivity [44]. To reduce observer subjectivity and standardize brain tumor classification, the WHO recently set new guidelines for diagnosis that include both molecular testing and histological examination [56]. Therefore, we would propose that the CI gene signature score be used in conjunction with histological examination as a diagnostic tool to determine tumor grade. Importantly, using the CI gene signature score as a diagnostic tool does not require isolating specific primary tumor cell populations (e.g. CSCs) given that the CI gene signature score is prognostic for tumor grade using data sets that performed bulk tumor cell gene expression profiling. In addition, a custom gene signature expression panel of the 269 copy number independent genes could be used to focus bioinformatics analysis efforts. Notably, any gene expression profiling approach reduces inter-observer subjectivity and variability to facilitate clinical diagnosis.

In addition to using the CI gene signature to assess tumor grade, we propose that patients could be stratified into CI-Hi and CI-Lo groups to inform clinical management of the disease. Patients in the CI-Lo group would potentially require more aggressive treatment plans as this group has a shorter survival time while patients in the CI-Hi group with longer survival times could potentially follow less intensive treatment regimens to minimize complications and adverse side effects. The use of the CI gene signature to inform treatment options will require additional studies to validate this approach. Ultimately, however, both patients in the CI-Hi and CI-Lo groups succumb to the disease illustrating the critical need for new therapeutic strategies to treat gliomas. Our differential gene expression analysis of NSCs and CSCs provides a list of potential candidates to selectively target CSCs over NSCs.

## Conclusions

In conclusion, we show that CIN is an extensive source of both genetic and transcriptional intra-tumor heterogeneity in chromosomally unstable cells as most gene expression scales proportionally to chromosome copy number. However, we also identify a subset of genes whose expression levels are regulated independent of copy number delineating a mechanism that buffers gene expression levels against the continual variability arising from a CIN phenotype. Moreover, from the genes whose expression levels are buffered against copy number alterations, we define a gene signature that predicts glioma tumor grade and patient survival which could help to inform clinical diagnosis and disease management.

## Methods

### Acquisition of single-cell RNA sequencing libraries

The CB660 NSCs and GliNS2 CSCs were washed and filtered with 20 μm strainer before loading into C1IFC. Live/DEAD solutions and single cell suspension were loaded following the C1 mRNA seq protocol. Each capture was examined under the microscope for viability and doublets. The cell lysing, RT and cDNA amplification were performed on C1 Single Cell Auto Prep system (Fluidigm). The cDNA was harvested only from the viable, single cells and Illumina sequencing library was constructed with Nextera XT DNA sample prep kit (Illumina).

### Pre-processing of single-cell RNA sequencing data

Beginning with 192 cells, cells with barcodes labeled as duplicates or no cell were filtered out, resulting in 155 cells. Of those 155 cells, cells with a low library size (library size < median library size – 3*median absolute deviation (MAD) of the library size distribution) or with a high fraction of mitochondrial genome (fraction of mitochondria genome > median fraction of mitochondria genome + 3*MAD of mitochondria genome distribution) were filtered out, resulting in 134 cells. Raw counts from the remaining cells were normalized using the *normalize* function from the “simpleSingleCell” R package [28].

The cell cycle phase of each single cell was computationally determined using previously published methods for identifying transcriptional cell cycle signatures [27, 28]. More specifically, cell cycle genes were constructed by identifying pairs of genes where the difference between gene expression within each pair correlated with the cell cycle phase. The cell cycle was then assigned by examining the difference of the gene pairs for each cell in our data. To do this, we used *cyclone* function from the “scran” R package for cell cycle inference [27].

### Chromosome copy number estimation

To analyze copy number variation, we used a method similar to the one reported by Patel et al. [29]. This method determines copy number using the average relative expression level of a sliding window of genomically-adjacent genes. To achieve an accurate estimation of the copy number variation, we first filtered out low abundance genes, defined as genes whose average expression is in the bottom 70th percentile, which may confound estimation analysis. Moreover, cells with a normalized library size < 26,000 counts (library size < median library size – 2*median absolute deviation (MAD) of the library size distribution) or a high fraction of zero counts (fraction of zero counts< 25%) in the remaining genes were filtered out. In total, 121 cells expressing 6350 genes were used for the analysis.

To create a reference normal profile, we took the geometric mean of each gene across all CB660 NSCs. We then calculated the relative gene expression of each single GliNS2 CSCs or CB660 NSCs as the log2 gene fold-change compared to the reference profile. Median normalization was used to normalize the relative gene expression profiles of each 121 cells. We then arranged each gene by their chromosomal coordinates and calculated the estimated copy number (ECN) of each gene *i* in cell *k* was calculated using the following formula [29]:

$$ EC{N}_k(i)=\frac{\sum_{j=i-50}^{i+50}{X}_{k(j)}}{101} $$

where *ECN*_*k*_(*i*) refers to the estimated copy number of cell *k* at gene *i* and *X*_*k*(*j*)_ refers to the relative gene expression of that gene in cell *k*. To minimize noisy copy number estimates, we forced all values where |*ECN*_*k*_(*i*)| < *log*_2_(0.5) to be zero.

To quantify the chromosome instability, we used the following formula:

$$ Chromosome\ instabilit{y}_k=\frac{\sum_{i=1}^n\left| EC{N}_k(i)\right|}{n} $$

where *Chromosome instability*_*k*_ refers to the chromosome instability of cell *k*, *ECN*_*k*_(*i*) refers to the estimated copy number of cell *k* at gene *i* and *n* refers to the total number of genes that were used for copy number estimation.

### Copy number independent signature and score calculation

We defined the copy number independent (CI) gene signature by comparing the differential expression of genes between GliNS2 CSCs and CB660 NSCs while adjusting for their estimated chromosome copy number. We imputed the copy number of genes lacking ECN values using the average of nearest genes’ estimated copy number. For each gene, we then constructed a logistic regression model using cell labels as the response variable (Y = 1 for GliNS2 cells, and Y = 0 for CB660 cells).

$$ \mathrm{In}\left(\frac{Y}{1-Y}\right)={\beta}_0+{\beta}_1\ast \kern0.62em \mathit{\exp}\kern0.5em +{\beta}_2\kern0.5em \ast \kern0.5em CNV $$

The predictor variables include expression level of the gene under consideration (*exp*) and that gene’s copy number. We used models to compare the copy number adjusted differential expression activity between GliNS2 CSCs and CB660 NSCs, and then estimated the coefficients (β-values) and the statistical significance (*p*-value) for all genes with corresponding Bonferroni-corrected statistical significance. We then used the β-values to separate the genes into up- and down-regulated gene sets, which were annotated as a pair of weight profiles, w^+^ and w^−^ for up and down respectively. For each weight profile, we forced genes exhibiting a significantly differential up- or down-regulated expression to equal 1 and forced insignificant genes to be 0. The resulting weight profiles define the magnitude by which each gene is differentially expressed in GliNS2 CSCs after adjusting for copy number. For example, if a gene *i* is more significantly up-regulated in GliNS2 CSCs versus CB660 NSCs, it will have a ${w}_i^{+}$of one and ${w}_i^{-}$ of zero. For down-regulated genes, the reverse will be true, with these genes having a ${w}_i^{-}$of 1 and a ${w}_i^{+}$of 0. To generate the CI score, we then integrated the sample-specific expression profiles of the glioma patients with the weight profiles, as previously described [59, 60]. We annotated the function of the CI signature using gene ontology (GO) enrichment analysis from the GO database.

### Public dataset collection

We used RNA-seq and copy number variation (CNV) data for breast invasive carcinoma (BRCA) and glioblastoma multiforme (GBM) samples generated by The Cancer Genome Atlas (TCGA) to validate the CNV estimation method. We downloaded Level 3 TCGA BRCA and GBM RNA-seq and CNV data from FireBrowse (gdac.broadinstitute.org/). The processed BRCA datasets consisted of gene expression profiles for a total of 1100 tumor samples and 112 normal samples and provided the RSEM-normalized expression and CNV for 20,502 genes. The processed GBM datasets consisted of gene expression profiles for a total of 161 tumor samples and 5 normal samples, and provided the RSEM-normalized expression and CNV for 20,502 genes. Also, we used microarray data for GBM samples generated by TCGA to examine the association between CI score and molecular subtypes, and these processed datasets consisted of gene expression profiles for a total of 539 samples and provided the lowess-normalized expression for 12,042 genes.

We used five additional microarray gene expression datasets to perform a series of analyses, including association analysis of the clinical factors and prediction of patient survival. Four datasets are available from the Gene Expression Omnibus (GEO) database under accession numbers GSE4271, GSE4412, GSE16011 and GSE1993. Sample sizes of these datasets are 77, 85, 263 and 65, respectively [42, 43, 46–48]. We also downloaded a dataset from a published paper by Nutt et al. [45] with a sample size of 50 (Additional file 8: Table S6).

### Statistical analysis

The R function *heatmap.2* was used to perform hierarchical clustering analyses. Principal component analysis was performed using the R function *prcomp*. To annotate the function of the CI signature, we performed the gene ontology (GO) enrichment analysis using the GO database. All analyses were performed under R version 3.4.3.

Survival regression was performed using Cox proportional hazard models to investigate the association between patient-specific CI score and patient overall survival. To perform two-class comparisons, samples were stratified using the median CI score. We then fitted a univariate Cox regression model to determine the association between the dichotomized CI score and patient survival. Log-rank tests were used to compare survival distributions between two groups.

All survival analyses were performed using the R “survival” package (3.4.3). Specifically, the “coxph” function was used to construct Cox proportional hazard models. The “survfit” function was used to generate a Kaplan-Meier survival curve for each group. The “survdiff” function was used to perform the log-rank test comparing the difference between survival curves.

## Additional files

Additional file 1: Single-cell RNA sequencing data normalization and filtering steps. a, Flowchart depicting data pre-processing steps. b, Graph showing the distribution of library sizes for all single cells. The red line indicates the cut-off used for filtering cells with low library size. c, Graph showing the distribution of the number of expressed genes in all single cells. The red line indicates the cut-off used for filtering cells with low numbers of expressed genes. d, Graph showing the distribution of mitochondrial genome in all single cells. The red line indicates the cut-off used for filtering cells with a high fraction of mitochondrial genome. e, Bar graphs showing the total counts distribution before and after normalization using four cells as examples. The green color indicates GliNS2 CSCs and the blue color indicates CB660 NSCs. Bar, median; box 25th to 75th percentile; whiskers, minimum and maximum. (TIF 9424 kb)

Additional file 2: Validation of estimated copy number in TCGA GBM dataset. a, Heatmap of estimated copy number (ECN) of all chromosomes (columns) in GBM cancer tissue and adjacent normal tissue (rows). On the scale, ECN = 0 indicates diploid gene expression levels. b, Quantification of chromosomal instability in tumor tissue and adjacent normal tissue. Bar, median; box 25th to 75th percentile; whiskers, minimum and maximum. *P* = 2E-4, Mann-Whitney U test comparing tumor and normal tissue. c, Correlation between ECN and SNP-array measured copy number using patient sample TCGA-12-0619-01A. Spearman correlation coefficient = 0.71. d, The distribution of the correlation coefficient across samples in the glioma dataset. The dashed line indicates the median correlation. (TIF 5482 kb)

Additional file 3: Validation of estimated copy number in TCGA BRCA dataset. a, Heatmap of estimated copy number (ECN) of all chromosomes (columns) in breast cancer tissue and adjacent normal tissue (rows). On the scale, ECN = 0 indicates diploid gene expression levels. b, Quantification of chromosomal instability in tumor tissue and adjacent normal tissue. Bar, median; box 25th to 75th percentile; whiskers, minimum and maximum. *P* < 2E-16, Mann-Whitney U test comparing tumor and normal tissue. c, Correlation between ECN and SNP-array measured copy number using patient sample TCGA-BH-A0DS-01A. Spearman correlation coefficient = 0.77. d, The distribution of the correlation coefficient across samples in BRCA dataset. The dashed line indicates the median correlation. (TIF 5896 kb)

Additional file 4: Estimated chromosome copy number analysis in GliNS2 CSCs and CB660 NSCs. a, The distribution of average normalized expression across cells (left) and the distribution of the total normalized counts of the 6350 genes across cells (right). The red line indicates the threshold used to filter out the unqualified genes or cells for estimated chromosome copy number analysis. b, Heatmap of shuffled ECN for all chromosomes (columns) in single CB660 NSCs and GliNS2 CSCs (rows). On the scale, ECN = 0 indicates diploid gene expression levels. The column adjacent to the heatmap shows the cell cycle phase of each cell as determined in Fig. 1b with the color of the bar corresponding to the predicted cell cycle phase. c, Quantification of chromosomal instability for shuffled ECN analysis in CB660 NSCs and GliNS2 CSCs. Bar, median; box 25th to 75th percentile; whiskers, minimum and maximum. *P* > 0.05; Mann-Whitney U test comparing CB660 NSCs and GliNS2 CSCs. d, Heatmap of ECN for all chromosomes (columns) in single GliNS2 CSCs (rows) using average gene expression in GliNS2 CSCs as the reference. On the scale, ECN = 0 indicates diploid gene expression levels. The column adjacent to the heatmap shows the cell cycle phase of each cell as determined in Fig. 1b with the color of the bar corresponding to the predicted cell cycle phase. (TIF 13786 kb)

Additional file 5: Validation of adjusting gene expression by ECN. a, The distribution of correlation coefficients between adjusted gene expression using SNP-array measured CNV and RNA-seq inferred CNV for TCGA GBM tumor samples (*n* = 17,949 genes). The median correlation is 0.92. b, Scatter plot showing the mean log2 fold change in expression for each differentially expressed gene before and after copy number adjustment. Red points indicate genes that remain significantly differentially expressed after copy number adjustment while blue points indicate genes that are not significantly differentially expressed after copy number adjustment. Spearman correlation coefficient = 0.93. (TIF 7342 kb)

Additional file 6: Gene enrichment analysis, growth rate of GliNS2 CSCs, and CI gene signature score. a, Gene ontology analysis of copy number adjusted genes with increased expression in GliNS2 CSCs compared to CB660 NSCs. Dashed line indicates an enrichment ratio = 1. b, Gene ontology analysis of copy number adjusted genes with decreased expression in GliNS2 CSCs compared to CB660 NSCs. Dashed line indicates an enrichment ratio = 1. c, The growth of GliNS2 CSCs was monitored every other day for 13 days total with an alamarBlue® assay. GliNS2 CSC population doubling time was calculated during the exponential phase of growth from Days 3–9. Three independent replicates were performed and error bars represent ±SD. d, Graph showing the copy number independent (CI) gene signature score for glioma samples in data set GSE16011 stratified by histological grade. Grade I/II = pilocytic astrocytoma (*n* = 8), astrocytoma (*n* = 13), oligodendroglial (n = 8) and mixed oligoastrocytic (*n* = 3), Grade III = astrocytoma (*n* = 16), oligodendroglial (*n* = 44) and mixed oligoastrocytic (*n* = 25), and Grade IV = glioblastoma (*n* = 159). Bar, median; box 25th to 75th percentile; whiskers, minimum and maximum. *P* = 7E-11, ANOVA analysis. e, Bar graph showing the proportions of grade III or grade IV tumors in the CI-Lo and CI-Hi groups for GSE4412. The exact percent of Grade III tumors in each group is indicated on top of the bars. *P* = 3E-4, Chi-square test comparing distribution between CI-Lo and CI-Hi groups. (TIF 17547 kb)

Additional file 7: CI gene signature is a novel gene signature in glioblastoma. a, Bar graph showing the average CI gene signature score across glioma CSCs (ALPS 1459), glioma non-CSCs (U87MG), and normal human astrocytes. The error bar is one standard deviation plus the CI score. *P* = 2E-3, ANOVA analysis. b, Bar graph showing the CI gene signature score across the four glioblastoma molecular subtypes classified in Verhaak et al. [41] using TCGA glioblastoma data. Pro = proneural, Neu = neural, Clas = classical, and Mese = mesenchymal. Bar, median; box 25th to 75th percentile; whiskers, minimum and maximum. *P* < 2E-16, ANOVA analysis. c, Heatmap showing the relative gene expression of the CI genes that do not overlap with the gene signature identified by Verhaak et al. [41] using TCGA glioblastoma data. (TIF 5435 kb)

Additional file 8: **Table S1**. Differentially expressed genes unadjusted for copy number. The column names refer to the T.score, T.test *p* value, Mann-Whitney U test p value, the log2 gene expression fold change and the average gene expression between GliNS2 and CB660 cells. **Table S2.** Copy number dependent differentially expressed genes. The column names that are labeled in green refer to the CNV unadjusted T.score, T.test p value, Mann-Whitney U test p value and the Bonferroni adjusted p value. The column names that are labeled in red refer to the CNV adjusted coefficient in the model, p value and adjusted p value. The column names that are labeled in blue refer to the pearson correlation coefficient between original gene expression and its estimated copy number, spearman correlation coefficient between original gene expression and its estimated copy number and the chromosome position of the genes. **Table S3.** Copy number independent differentially expressed genes. The column names that are labeled in green refer to the CNV unadjusted T.score, T.test p value, Mann-Whitney U test p value and the Bonferroni adjusted p value. The column names that are labeled in red refer to the CNV adjusted coefficient in the model, p value and adjusted *p* value. The column names that are labeled in blue refer to the pearson correlation coefficient between original gene expression and its estimated copy number, spearman correlation coefficient between original gene expression and its estimated copy number and the chromosome position of the genes. **Table S4.** Copy number adjusted differentially expressed genes enrichment. Gene ontology enrichment analysis of the CI genes. The column names refer to the gene ontology (GO) term, the number of genes in the GO term, the number of overlapped genes between CI genes and the GO term, the enrichment ratio of the GO term, the statistical significance of the enrichment (p value) and the statistical significance of the enrichment after multiple testing correction (p.adjust). **Table S5.** Genes enriched in negative regulation of cell cycle. The column names refer to the coefficient of the gene in the copy number adjusted model, the p value of each gene after copy number adjustment, the log2 gene fold change between GliNS2 and CB660 cells, the average gene expression between GliNS2 and CB660 cells, the Pearson and Spearman correlation between original gene expression and copy number variation, the position of each gene on the chromosome, the GO term ID and GO term name. **Table S6.** Dataset summary. Sample sizes for the five additional microarray gene expression datasets used to perform association analysis of clinical factors and prediction of patient survival. (XLSX 434 kb)

## Abbreviations

BRCA: Breast invasive carcinoma
CI: Copy number independent
CIN: Chromosomal instability
CNV: Copy number variation
CSC: Cancer stem cell
ECN: Estimated copy number
GBM: Glioblastoma multiforme
GEO: Gene Expression Omnibus
GO: Gene ontology
MAD: Median absolute deviation
NSC: Neural stem cell
PCA: Principal component analysis
SCC: Spearman correlation coefficient
TCGA: The Cancer Genome Atlas
WHO: The World Health Organization

## References

[1] Burrell RA, Swanton C (2014). Tumour heterogeneity and the evolution of polyclonal drug resistance. Mol Oncol.

[2] Burrell RA, McGranahan N, Bartek J, Swanton C (2013). The causes and consequences of genetic heterogeneity in cancer evolution. Nature..

[3] Meacham CE, Morrison SJ (2013). Tumour heterogeneity and cancer cell plasticity. Nature..

[4] Visvader JE, Lindeman GJ (2008). Cancer stem cells in solid tumours: accumulating evidence and unresolved questions. Nat Rev Cancer.

[5] Bao S, Wu Q, McLendon RE, Hao Y, Shi Q, Hjelmeland AB (2006). Glioma stem cells promote radioresistance by preferential activation of the DNA damage response. Nature..

[6] Oravecz-Wilson KI, Philips ST, Yilmaz ÖH, Ames HM, Li L, Crawford BD (2009). Persistence of leukemia-initiating cells in a conditional Knockin model of an Imatinib-responsive myeloproliferative disorder. Cancer Cell.

[7] Orr B, Godek KM, Compton D (2015). Aneuploidy. Curr Biol.

[8] Weaver BA, Cleveland DW (2006). Does aneuploidy cause cancer?. Curr Opin Cell Biol.

[9] Godek KM, Venere M, Wu Q, Mills KD, Hickey WF, Rich JN (2016). Chromosomal instability affects the Tumorigenicity of glioblastoma tumor-initiating cells. Cancer Discovery.

[10] Lee A, Endesfelder D, Rowan AJ, Walther A, Birkbak NJ, Futreal AP (2011). Chromosomal instability confers intrinsic multidrug resistance. Cancer Res.

[11] Bakhoum SF, Danilova OV, Kaur P, Levy NB, Compton DA (2011). Chromosomal instability substantiates poor prognosis in patients with diffuse large B-cell lymphoma. Clin Cancer Res.

[12] Orr B, Talje L, Liu Z, Kwok BH, Compton DA (2016). Adaptive resistance to an inhibitor of chromosomal instability in human Cancer cells. Cell Rep.

[13] Stranger BE, Forrest MS, Dunning M, Ingle CE, Beazley C, Thorne N (2007). Relative impact of nucleotide and copy number variation on gene expression phenotypes. Science..

[14] Torres EM, Sokolsky T, Tucker CM, Chan LY, Boselli M, Dunham MJ (2007). Effects of aneuploidy on cellular physiology and cell division in haploid yeast. Science..

[15] Williams BR, Prabhu VR, Hunter KE, Glazier CM, Whittaker CA, Housman DE (2008). Aneuploidy affects proliferation and spontaneous immortalization in mammalian cells. Science..

[16] Stingele S, Stoehr G, Peplowska K, Cox J, Mann M, Storchova Z. Global analysis of genome, transcriptome and proteome reveals the response to aneuploidy in human cells. Mol Syst Biol. 2012;8. 10.1038/msb.2012.40 PMC3472693 22968442

[17] Pavelka N, Rancati G, Zhu J, Bradford WD, Saraf A, Florens L (2010). Aneuploidy confers quantitative proteome changes and phenotypic variation in budding yeast. Nature..

[18] Pollack JR, Sørlie T, Perou CM, Rees CA, Jeffrey SS, Lonning PE (2002). Microarray analysis reveals a major direct role of DNA copy number alteration in the transcriptional program of human breast tumors. Proc National Acad Sci.

[19] Sun Y, Pollard S, Conti L, Toselli M, Biella G, Parkin G (2008). Long-term tripotent differentiation capacity of human neural stem (NS) cells in adherent culture. Mol Cell Neurosci.

[20] Pollard SM, Yoshikawa K, Clarke ID, Danovi D, Stricker S, Russell R (2009). Glioma stem cell lines expanded in adherent culture have tumor-specific phenotypes and are suitable for chemical and genetic screens. Cell Stem Cell.

[21] Stupp R, Hegi ME, Mason WP, van den Bent MJ, Taphoorn MJ, Janzer RC (2009). Effects of radiotherapy with concomitant and adjuvant temozolomide versus radiotherapy alone on survival in glioblastoma in a randomised phase III study: 5-year analysis of the EORTC-NCIC trial. The Lancet Oncology.

[22] Chen J, Li Y, Yu T-S, McKay RM, Burns DK, Kernie SG (2012). A restricted cell population propagates glioblastoma growth after chemotherapy. Nature..

[23] Galli R, Binda E, Orfanelli U, Cipelletti B, Gritti A, Vitis S (2004). Isolation and characterization of tumorigenic, stem-like neural precursors from human glioblastoma. Cancer Res.

[24] Lathia JD, Gallagher J, Myers JT, Li M, Vasanji A, McLendon RE (2011). Direct in vivo evidence for tumor propagation by glioblastoma Cancer stem cells. PLoS One.

[25] Singh S, Clarke I, Terasaki M, Bonn V, research HC (2003). Identification of a cancer stem cell in human brain tumors.

[26] Engström PG, Tommei D, Stricker SH, Ender C, Pollard SM, Bertone P (2012). Digital transcriptome profiling of normal and glioblastoma-derived neural stem cells identifies genes associated with patient survival. Genome Medicine.

[27] Scialdone A, Natarajan KN, Saraiva LR, Proserpio V, Teichmann SA, Stegle O (2015). Computational assignment of cell-cycle stage from single-cell transcriptome data. Methods..

[28] Lun A, McCarthy DJ, Marioni JC (2016). A step-by-step workflow for low-level analysis of single-cell RNA-seq data. F1000Research..

[29] Patel AP, Tirosh I, Trombetta JJ, Shalek AK, Gillespie SM, Wakimoto H (2014). Single-cell RNA-seq highlights intratumoral heterogeneity in primary glioblastoma. Science..

[30] Taylor AM, Shih J, Ha G, Gao GF, Zhang X, Berger AC, et al. Genomic and functional approaches to understanding Cancer aneuploidy. Cancer Cell. 2018. 10.1016/j.ccell.2018.03.007 PMC6028190 29622463

[31] Wiltshire Rodney N., Rasheed B.K. Ahmed, Friedman Henry S., Friedman Allan H., Bigner Sandra H. (2000). Comparative genetic patterns of glioblastoma multiforme: Potential diagnostic tool for tumor classification. Neuro-Oncology.

[32] Baronchelli S, Bentivegna A, Redaelli S, Riva G, Butta V, Paoletta L (2013). Delineating the Cytogenomic and Epigenomic landscapes of glioma stem cell lines. PLoS One.

[33] Hose J, Yong C, Sardi M, Wang Z, Newton MA, Gasch AP (2015). Dosage compensation can buffer copy-number variation in wild yeast. eLife..

[34] Surana R, Sikka S, Cai W, Shin E, Warrier SR, Tan H (2014). Secreted frizzled related proteins: implications in cancers. Biochimica et Biophysica Acta (BBA) - Reviews on Cancer.

[35] Julien SG, Dubé N, Hardy S, Tremblay ML (2011). Inside the human cancer tyrosine phosphatome. Nat Rev Cancer.

[36] Lahoz A, Hall A (2008). DLC1: a significant GAP in the cancer genome. Genes Dev.

[37] Chen W, Dong J, Haiech J, Kilhoffer M-C, Zeniou M (2016). Cancer stem cell quiescence and plasticity as major challenges in Cancer therapy. Stem Cells Int.

[38] Ke L, Shi Y, Im S, Chen X, Yung W (2000). The relevance of cell proliferation, vascular endothelial growth factor, and basic fibroblast growth factor production to angiogenesis and tumorigenicity in human glioma cell lines. Clin Cancer Res Official J Am Assoc Cancer Res.

[39] Vastrad B, Vastrad C, Godavarthi A, Chandrashekar R (2017). Molecular mechanisms underlying gliomas and glioblastoma pathogenesis revealed by bioinformatics analysis of microarray data. Med Oncol.

[40] Li F, Liu X, Sampson JH, Bigner DD, Li C-Y (2016). Rapid reprogramming of primary human astrocytes into potent tumor-initiating cells with defined genetic factors. Cancer Res.

[41] Verhaak R, Hoadley KA, Purdom E, Wang V, Qi Y, Wilkerson MD (2010). Integrated genomic analysis identifies clinically relevant subtypes of glioblastoma characterized by abnormalities in PDGFRA, IDH1, EGFR, and NF1. Cancer Cell.

[42] Petalidis LP, Oulas A, Backlund M, Wayland MT, Liu L, Plant K (2008). Improved grading and survival prediction of human astrocytic brain tumors by artificial neural network analysis of gene expression microarray data. Mol Cancer Ther.

[43] Gravendeel L, Kouwenhoven M, Gevaert O, de Rooi JJ, Stubbs AP, Duijm EJ (2009). Intrinsic gene expression profiles of gliomas are a better predictor of survival than histology. Cancer Res.

[44] Vigneswaran K, of translational … NS. Beyond the World Health Organization grading of infiltrating gliomas: advances in the molecular genetics of glioma classification. 2015. 10.3978/j.issn.2305-5839.2015.03.57 PMC4430738 26015937

[45] Nutt CL, Mani D, Betensky RA, Tamayo P, Cairncross GJ, Ladd C (2003). Gene expression-based classification of malignant gliomas correlates better with survival than histological classification. Cancer Res.

[46] Freije WA, Castro-Vargas EF, Fang Z, Horvath S, Cloughesy T, Liau LM (2004). Gene expression profiling of gliomas strongly predicts survival. Cancer Res.

[47] Phillips HS, Kharbanda S, Chen R, Forrest WF, Soriano RH, Wu TD (2006). Molecular subclasses of high-grade glioma predict prognosis, delineate a pattern of disease progression, and resemble stages in neurogenesis. Cancer Cell.

[48] Costa BM, Smith JS, Chen Y, Chen J, Phillips HS, Aldape KD (2010). Reversing HOXA9 oncogene activation by PI3K inhibition: epigenetic mechanism and prognostic significance in human glioblastoma. Cancer Res.

[49] Davoli T, Xu A, Mengwasser KE, Sack LM, Yoon JC, Park PJ (2013). Cumulative Haploinsufficiency and Triplosensitivity drive aneuploidy patterns and shape the Cancer genome. Cell..

[50] Kops GJ, Foltz DR, Cleveland DW (2004). Lethality to human cancer cells through massive chromosome loss by inhibition of the mitotic checkpoint. Proc Natl Acad Sci U S A.

[51] Janssen A, Kops GJ, Medema RH (2009). Elevating the frequency of chromosome mis-segregation as a strategy to kill tumor cells. Proc Natl Acad Sci.

[52] Silk AD, Zasadil LM, Holland AJ, Vitre B, Cleveland DW, Weaver BA (2013). Chromosome missegregation rate predicts whether aneuploidy will promote or suppress tumors. Proc Natl Acad Sci.

[53] Weaver B, Silk AD, Montagna C, Verdier-Pinard P, Cleveland DW (2007). Aneuploidy acts both Oncogenically and as a tumor suppressor. Cancer Cell.

[54] Dephoure N, Hwang S, O’Sullivan C, Dodgson SE, Gygi SP, Amon A (2014). Quantitative proteomic analysis reveals posttranslational responses to aneuploidy in yeast. eLife..

[55] Gusyatiner Olga, Hegi Monika E. (2018). Glioma epigenetics: From subclassification to novel treatment options. Seminars in Cancer Biology.

[56] Louis DN, Perry A, Reifenberger G, von Deimling A, Figarella-Branger D, Cavenee WK (2016). The 2016 World Health Organization classification of tumors of the central nervous system: a summary. Acta Neuropathol.

[57] Singh SK, Hawkins C, Clarke ID, Squire JA, Bayani J, Hide T (2004). Identification of human brain tumour initiating cells. Nature..

[58] Cavaliere R, Lopes MS, Schiff D (2005). Low-grade gliomas: an update on pathology and therapy. The Lancet Neurology.

[59] Zhao Yanding, Varn Frederick S., Cai Guoshuai, Xiao Feifei, Amos Christopher I., Cheng Chao (2017). A P53-Deficiency Gene Signature Predicts Recurrence Risk of Patients with Early-Stage Lung Adenocarcinoma. Cancer Epidemiology Biomarkers & Prevention.

[60] Cheng C, Yan X, Sun F, Li LM (2007). Inferring activity changes of transcription factors by binding association with sorted expression profiles. Bmc Bioinformatics.

