# Supplementary figures and images for: Single-cell RNA sequencing reveals the impact of chromosomal instability on glioblastoma cancer stem cells
Source: BMC Med Genomics. 2019 May 31;12:79. doi: 10.1186/s12920-019-0532-5 (PMC6545015; doi:10.1186/s12920-019-0532-5)

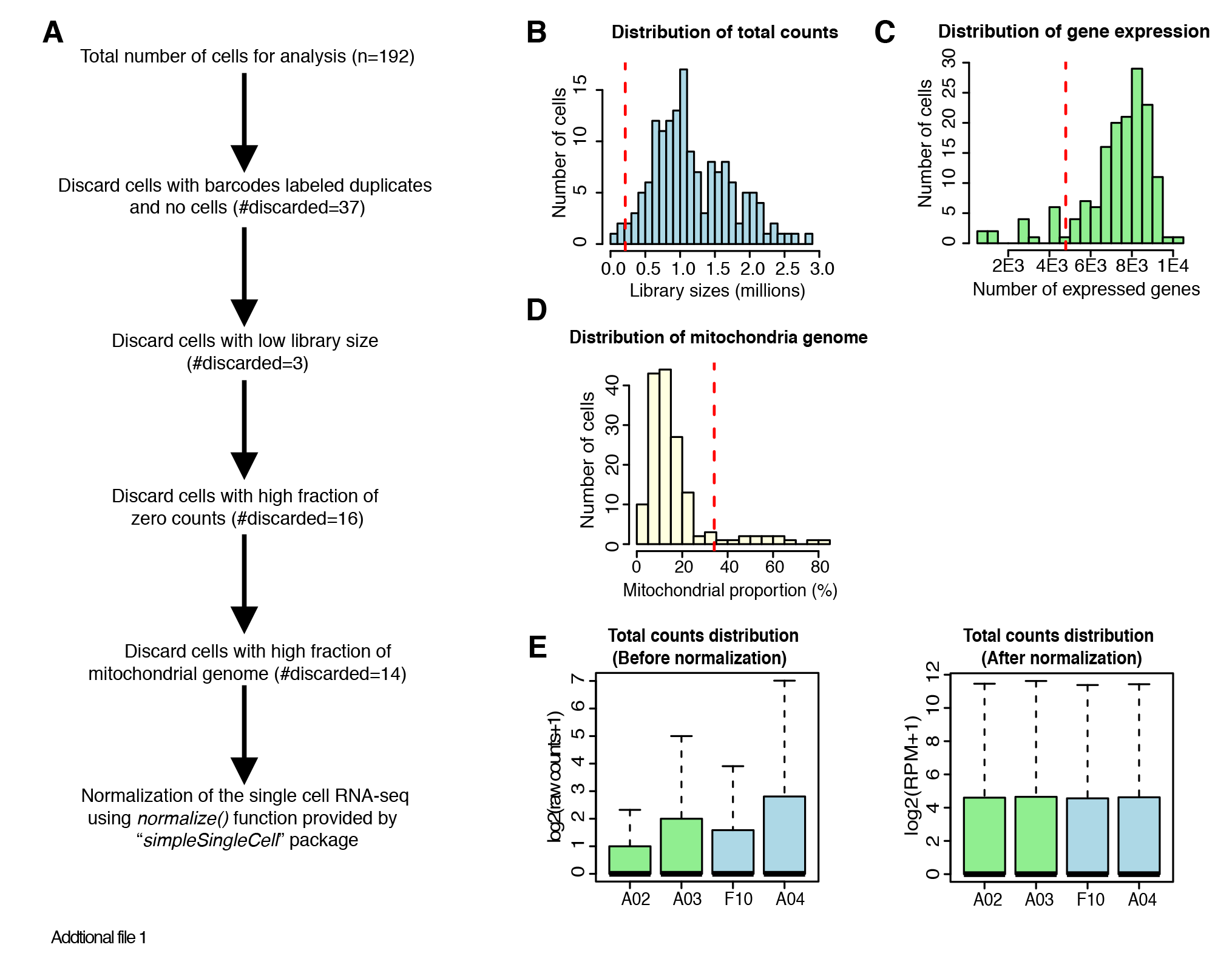

Supplement: Supplementary file 1 — Single-cell RNA sequencing data normalization and filtering steps. a, Flowchart depicting data pre-processing steps. b, Graph showing the distribution of library sizes for all single cells. The red line indicates the cut-off used for filtering cells with low library size. c, Graph showing the distribution of the number of expressed genes in all single cells. The red line indicates the cut-off used for filtering cells with low numbers of expressed genes. d, Graph showing the distribution of mitochondrial genome in all single cells. The red line indicates the cut-off used for filtering cells with a high fraction of mitochondrial genome. e, Bar graphs showing the total counts distribution before and after normalization using four cells as examples. The green color indicates GliNS2 CSCs and the blue color indicates CB660 NSCs. Bar, median; box 25th to 75th percentile; whiskers, minimum and maximum. (TIF 9424 kb) [file 12920_2019_532_MOESM1_ESM.tif]

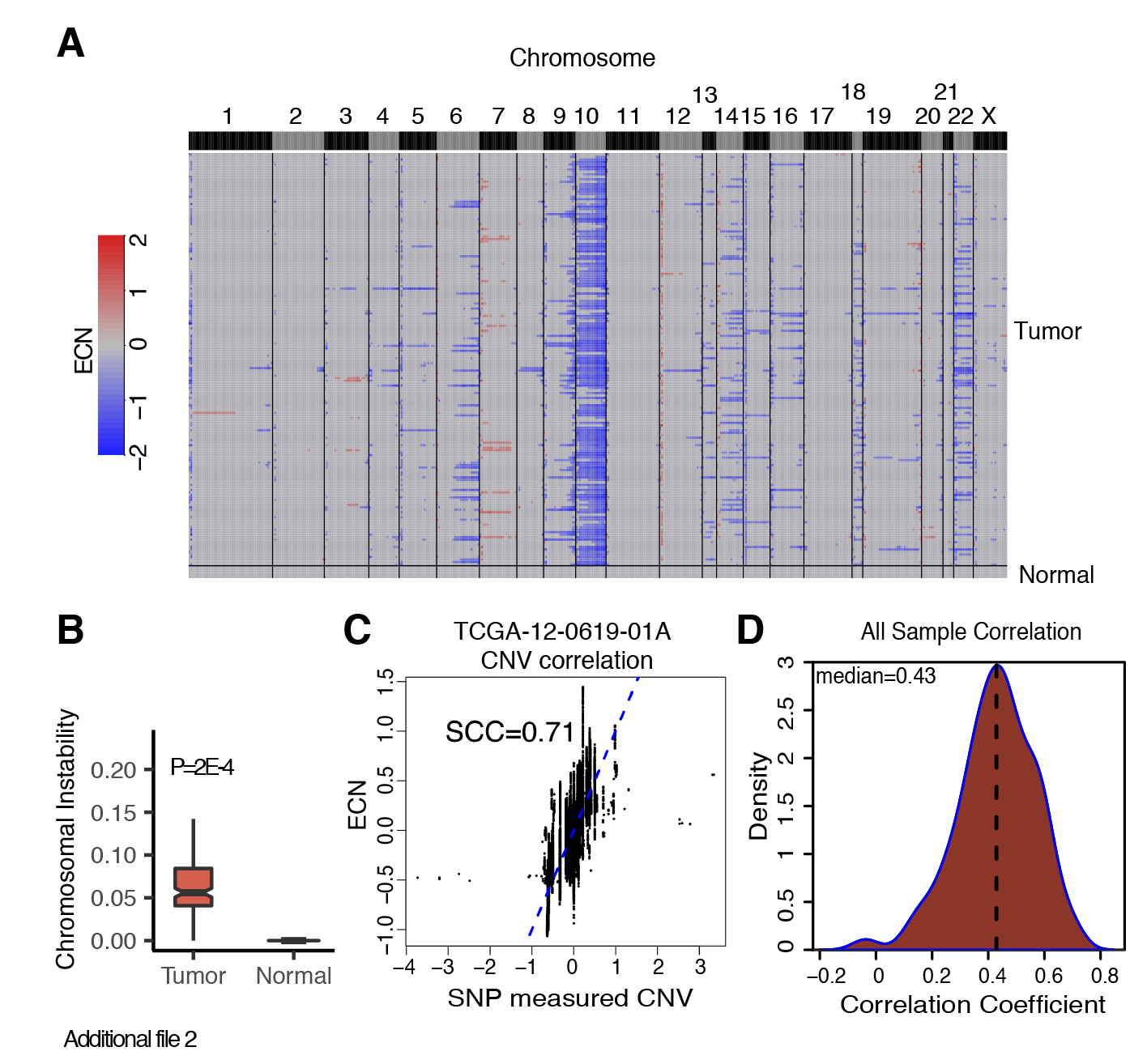

Supplement: Supplementary file 2 — Validation of estimated copy number in TCGA GBM dataset. a, Heatmap of estimated copy number (ECN) of all chromosomes (columns) in GBM cancer tissue and adjacent normal tissue (rows). On the scale, ECN = 0 indicates diploid gene expression levels. b, Quantification of chromosomal instability in tumor tissue and adjacent normal tissue. Bar, median; box 25th to 75th percentile; whiskers, minimum and maximum. P = 2E-4, Mann-Whitney U test comparing tumor and normal tissue. c, Correlation between ECN and SNP-array measured copy number using patient sample TCGA-12-0619-01A. Spearman correlation coefficient = 0.71. d, The distribution of the correlation coefficient across samples in the glioma dataset. The dashed line indicates the median correlation. (TIF 5482 kb) [file 12920_2019_532_MOESM2_ESM.tif]

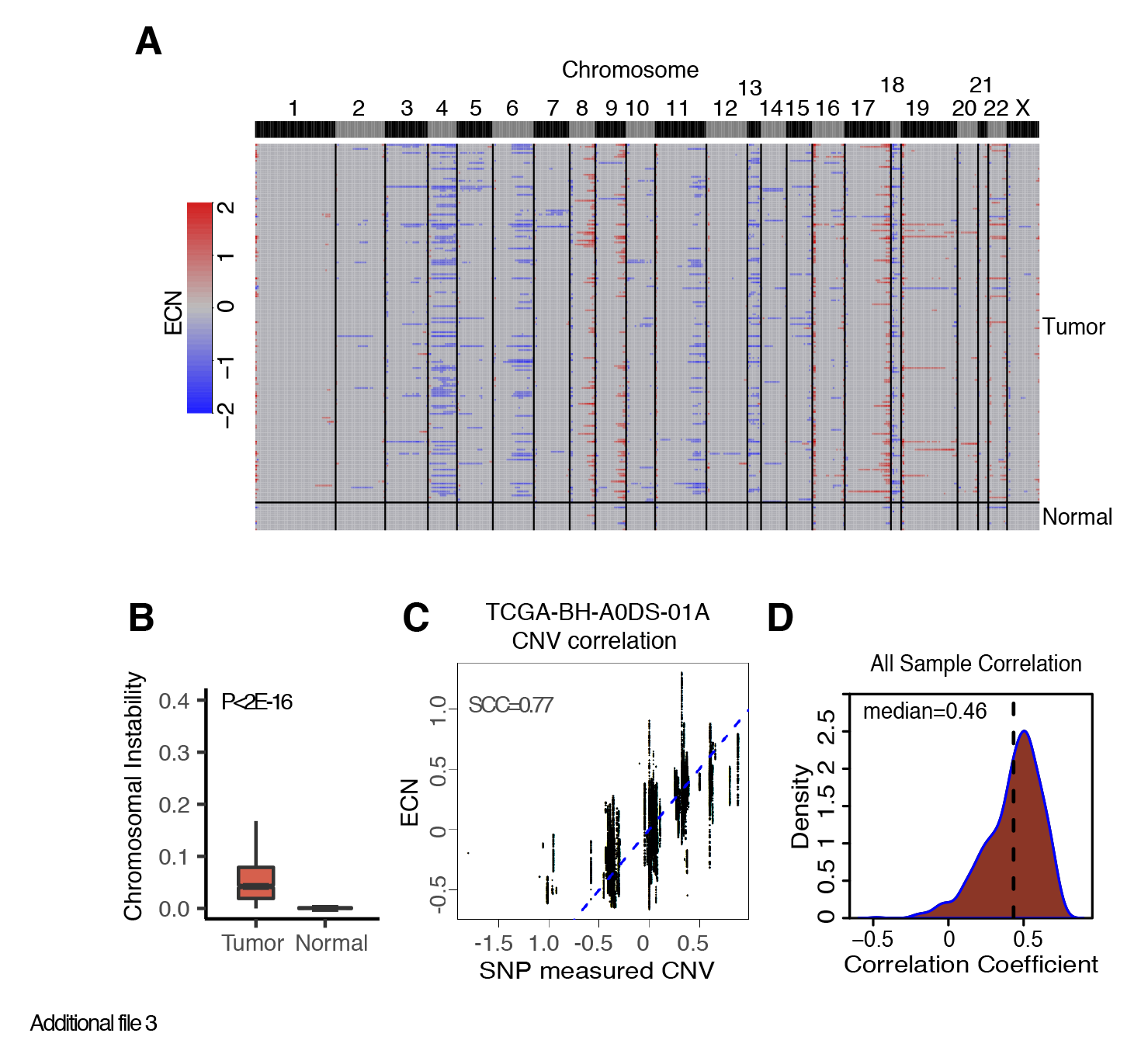

Supplement: Supplementary file 3 — Validation of estimated copy number in TCGA BRCA dataset. a, Heatmap of estimated copy number (ECN) of all chromosomes (columns) in breast cancer tissue and adjacent normal tissue (rows). On the scale, ECN = 0 indicates diploid gene expression levels. b, Quantification of chromosomal instability in tumor tissue and adjacent normal tissue. Bar, median; box 25th to 75th percentile; whiskers, minimum and maximum. P < 2E-16, Mann-Whitney U test comparing tumor and normal tissue. c, Correlation between ECN and SNP-array measured copy number using patient sample TCGA-BH-A0DS-01A. Spearman correlation coefficient = 0.77. d, The distribution of the correlation coefficient across samples in BRCA dataset. The dashed line indicates the median correlation. (TIF 5896 kb) [file 12920_2019_532_MOESM3_ESM.tif]

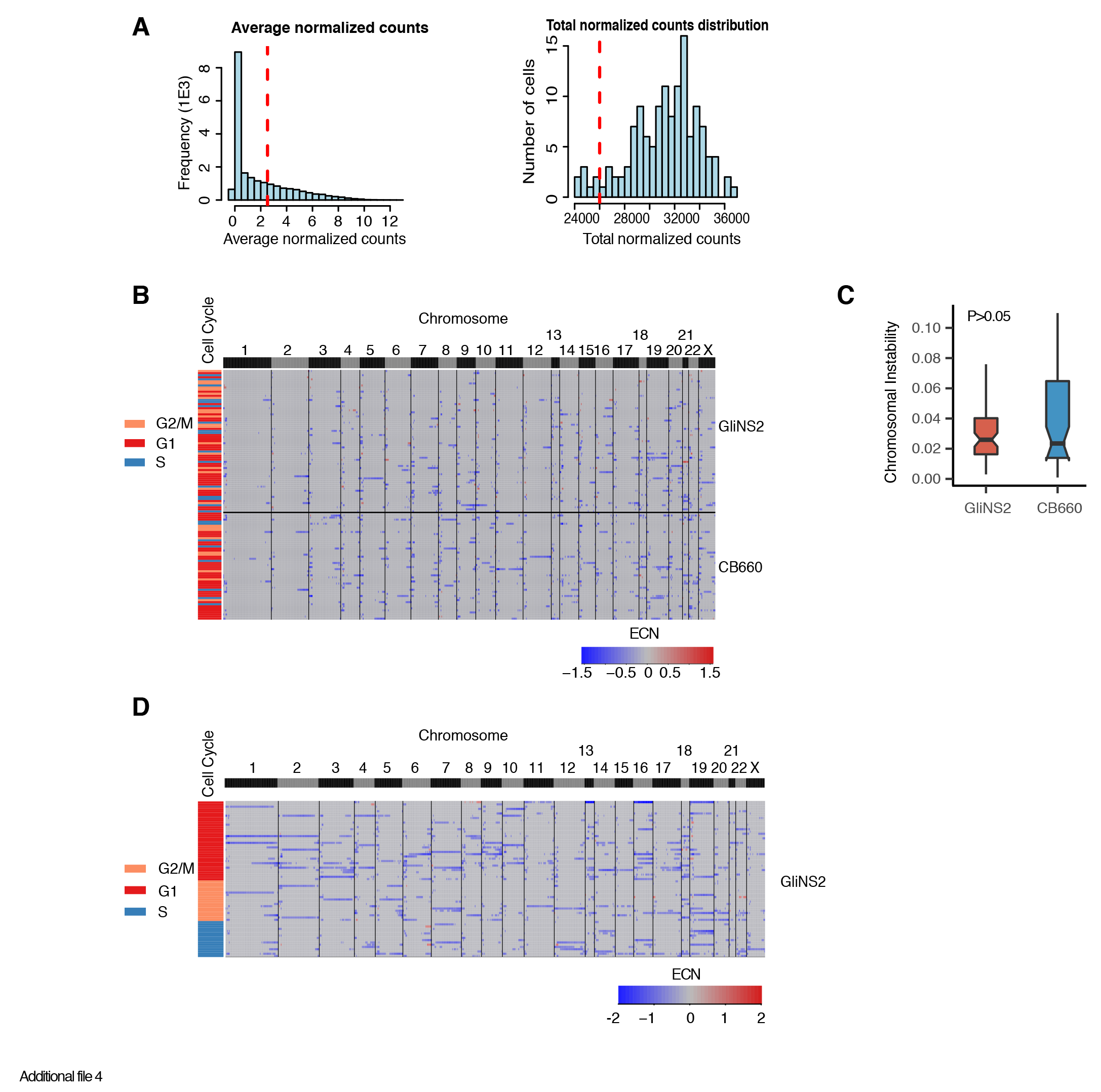

Supplement: Supplementary file 4 — Estimated chromosome copy number analysis in GliNS2 CSCs and CB660 NSCs. a, The distribution of average normalized expression across cells (left) and the distribution of the total normalized counts of the 6350 genes across cells (right). The red line indicates the threshold used to filter out the unqualified genes or cells for estimated chromosome copy number analysis. b, Heatmap of shuffled ECN for all chromosomes (columns) in single CB660 NSCs and GliNS2 CSCs (rows). On the scale, ECN = 0 indicates diploid gene expression levels. The column adjacent to the heatmap shows the cell cycle phase of each cell as determined in Fig. 1b with the color of the bar corresponding to the predicted cell cycle phase. c, Quantification of chromosomal instability for shuffled ECN analysis in CB660 NSCs and GliNS2 CSCs. Bar, median; box 25th to 75th percentile; whiskers, minimum and maximum. P > 0.05; Mann-Whitney U test comparing CB660 NSCs and GliNS2 CSCs. d, Heatmap of ECN for all chromosomes (columns) in single GliNS2 CSCs (rows) using average gene expression in GliNS2 CSCs as the reference. On the scale, ECN = 0 indicates diploid gene expression levels. The column adjacent to the heatmap shows the cell cycle phase of each cell as determined in Fig. 1b with the color of the bar corresponding to the predicted cell cycle phase. (TIF 13786 kb) [file 12920_2019_532_MOESM4_ESM.tif]

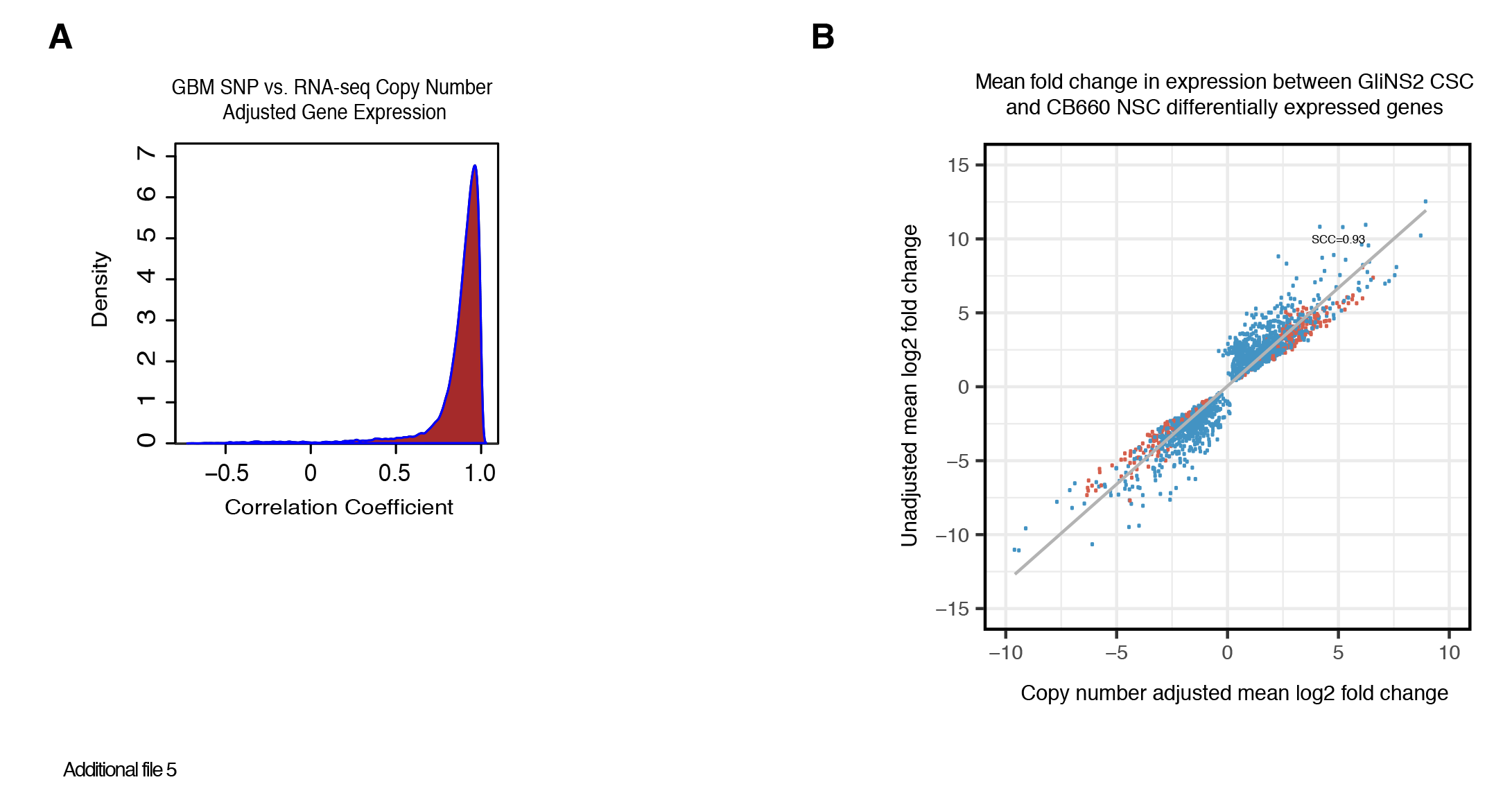

Supplement: Supplementary file 5 — Validation of adjusting gene expression by ECN. a, The distribution of correlation coefficients between adjusted gene expression using SNP-array measured CNV and RNA-seq inferred CNV for TCGA GBM tumor samples (n = 17,949 genes). The median correlation is 0.92. b, Scatter plot showing the mean log2 fold change in expression for each differentially expressed gene before and after copy number adjustment. Red points indicate genes that remain significantly differentially expressed after copy number adjustment while blue points indicate genes that are not significantly differentially expressed after copy number adjustment. Spearman correlation coefficient = 0.93. (TIF 7342 kb) [file 12920_2019_532_MOESM5_ESM.tif]

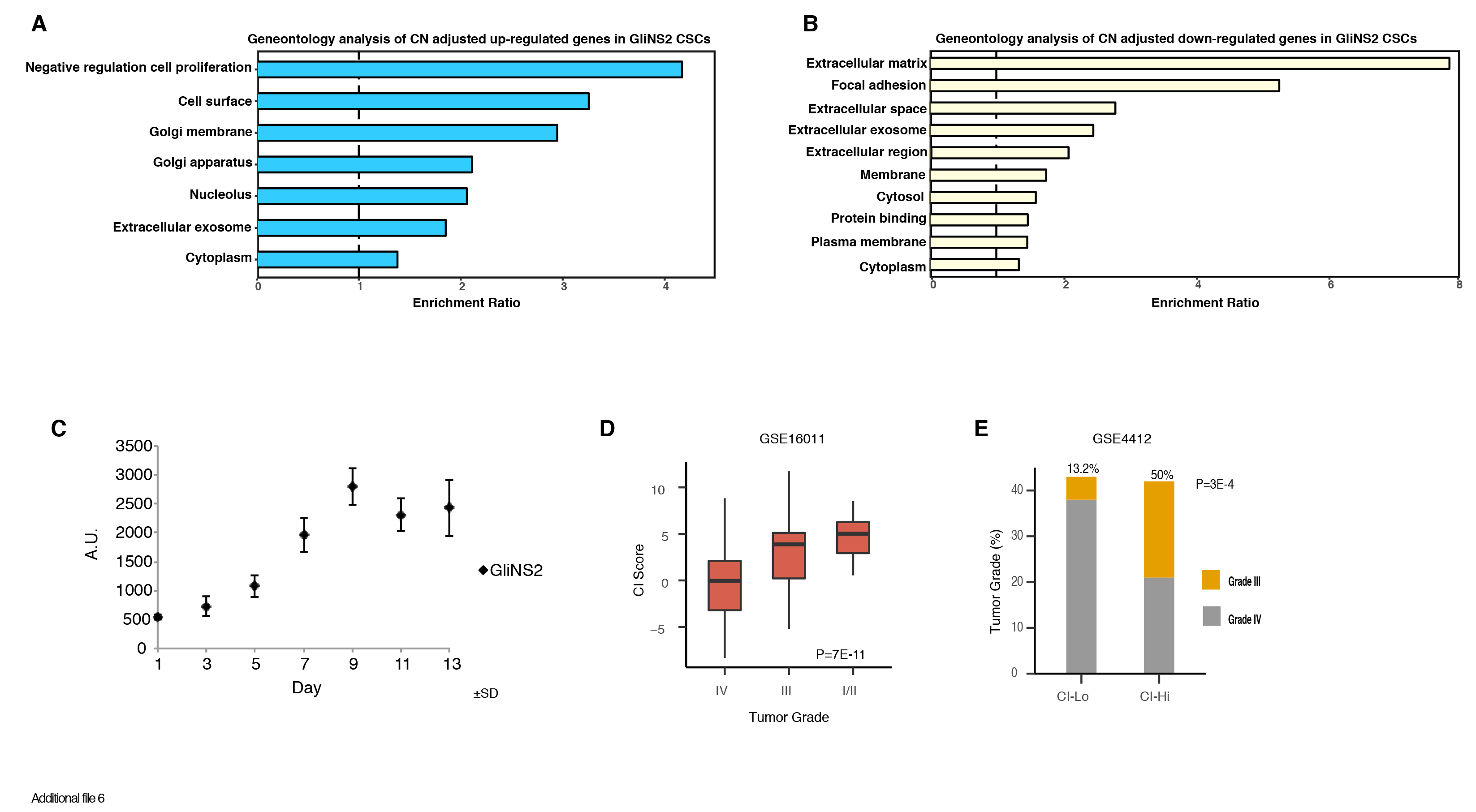

Supplement: Supplementary file 6 — Gene enrichment analysis, growth rate of GliNS2 CSCs, and CI gene signature score. a, Gene ontology analysis of copy number adjusted genes with increased expression in GliNS2 CSCs compared to CB660 NSCs. Dashed line indicates an enrichment ratio = 1. b, Gene ontology analysis of copy number adjusted genes with decreased expression in GliNS2 CSCs compared to CB660 NSCs. Dashed line indicates an enrichment ratio = 1. c, The growth of GliNS2 CSCs was monitored every other day for 13 days total with an alamarBlue® assay. GliNS2 CSC population doubling time was calculated during the exponential phase of growth from Days 3–9. Three independent replicates were performed and error bars represent ±SD. d, Graph showing the copy number independent (CI) gene signature score for glioma samples in data set GSE16011 stratified by histological grade. Grade I/II = pilocytic astrocytoma (n = 8), astrocytoma (n = 13), oligodendroglial (n = 8) and mixed oligoastrocytic (n = 3), Grade III = astrocytoma (n = 16), oligodendroglial (n = 44) and mixed oligoastrocytic (n = 25), and Grade IV = glioblastoma (n = 159). Bar, median; box 25th to 75th percentile; whiskers, minimum and maximum. P = 7E-11, ANOVA analysis. e, Bar graph showing the proportions of grade III or grade IV tumors in the CI-Lo and CI-Hi groups for GSE4412. The exact percent of Grade III tumors in each group is indicated on top of the bars. P = 3E-4, Chi-square test comparing distribution between CI-Lo and CI-Hi groups. (TIF 17547 kb) [file 12920_2019_532_MOESM6_ESM.tif]

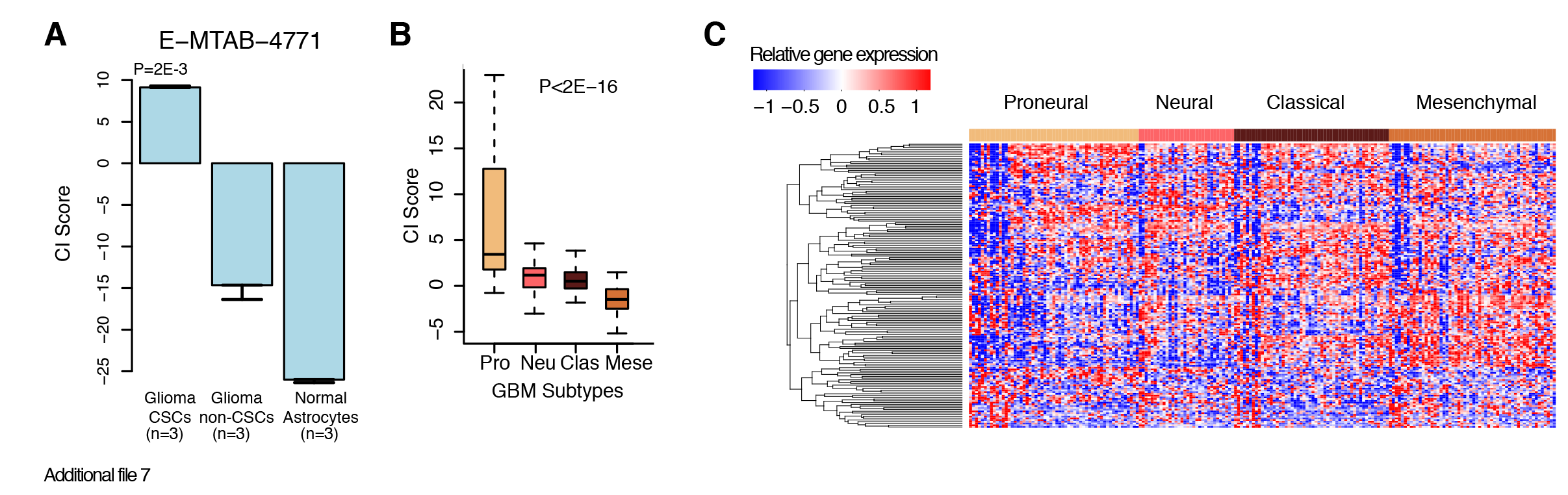

Supplement: Supplementary file 7 — CI gene signature is a novel gene signature in glioblastoma. a, Bar graph showing the average CI gene signature score across glioma CSCs (ALPS 1459), glioma non-CSCs (U87MG), and normal human astrocytes. The error bar is one standard deviation plus the CI score. P = 2E-3, ANOVA analysis. b, Bar graph showing the CI gene signature score across the four glioblastoma molecular subtypes classified in Verhaak et al. [41] using TCGA glioblastoma data. Pro = proneural, Neu = neural, Clas = classical, and Mese = mesenchymal. Bar, median; box 25th to 75th percentile; whiskers, minimum and maximum. P < 2E-16, ANOVA analysis. c, Heatmap showing the relative gene expression of the CI genes that do not overlap with the gene signature identified by Verhaak et al. [41] using TCGA glioblastoma data. (TIF 5435 kb) [file 12920_2019_532_MOESM7_ESM.tif]
